# Application and Modification of Nutritional Assessment Tools in Hematologic Malignancies

**DOI:** 10.3390/cancers18050765

**Published:** 2026-02-27

**Authors:** Xinying Chen, Xin Zheng, Chenan Liu, Qibiao Shi, Xiaoyue Liu, Zhaoting Bu, Hong Zhao, Bing Yin, Changhong Xu, Hanping Shi

**Affiliations:** 1 Department of Gastrointestinal Surgery/Department of Clinical Nutrition, Beijing Shijitan Hospital, Capital Medical University, Beijing 100038, China; 2 National Clinical Research Center for Geriatric Diseases, Xuanwu Hospital, Capital Medical University, Beijing 100053, China; 3 Beijing International Science and Technology Cooperation Base for Cancer Metabolism and Nutrition, Beijing 100038, China; 4 Key Laboratory of Cancer FSMP for State Market Regulation, Beijing 100038, China; 5 Longgang Yabeien Stomatological Clinic Co., Ltd., Wenzhou 325802, China

**Keywords:** nutrition, prognosis, hematologic malignancies, leukemia, lymphoma, overall survival

## Abstract

**Background:** Hematologic malignancies pose a critical threat to global health, with their pathological progression intrinsically linked to metabolic dysregulation and nutrient imbalance. Malnutrition accelerates the trajectory of adverse outcomes while substantially diminishing the quality of survival. Although several nutritional assessment tools are currently used in clinical practice, a significant evidence gap persists regarding their validation in populations with hematologic neoplasms. This study systematically evaluates the prognostic performance of existing nutritional assessment instruments in this cohort. Based on these findings, we further explored the feasibility of a preliminary framework that reflects metabolic characteristics specific to this population. **Methods:** This prospective cohort study analyzed nutritional assessment data from 1067 patients with hematologic malignancies enrolled in the INSCOC registry. Eight assessment systems were examined: Patient-Generated Subjective Global Assessment (PG-SGA), Modified PGSGA (mPG-SGA), PGSGA Short Form (PG-SGA SF), Abbreviated PGSGA (abPG-SGA), Nutritional Risk Screening-2002 (NRS-2002), Global Leadership Initiative on Malnutrition criteria (GLIM), Scored-GLIM, and Karnofsky Performance Status Scale (KPS). Kaplan–Meier survival curves and multivariate Cox regression analyses were conducted to investigate the association between nutritional status and overall survival (OS) and to determine the prognostic weight of individual components within the nutritional assessment tools. Linear regression models were applied to examine the relationships between nutritional assessment tools, length of hospital stay (LOS), and EORTC QLQ-C30 scores. The predictive performance of the tools was evaluated using the area under the receiver operating characteristic curve (AUC) and the concordance index (C-index). Least absolute shrinkage and selection operator (LASSO) regression was applied to optimize the selection of inflammation-related biomarkers. **Results:** A total of 1067 participants were analyzed (mean [SD] age, 55.54 [17.4] years; 625 were male [58.6%]). Cox proportional hazards regression demonstrated statistically significant associations for all eight nutritional assessment tools (*p* ≤ 0.05). However, their prognostic discrimination was limited, as indicated by the AUC analysis. Specifically, the area under the curve (AUC) values for each tool were as follows: mPG-SGA, 0.561; NRS-2002, 0.557; PG-SGA, 0.550; KPS, 0.544; PG-SGA SF, 0.542; abPG-SGA, 0.528; Scored-GLIM, 0.489; and GLIM, 0.473. The concordance index validation further corroborated these findings. Prognostically significant components and inflammation-related biomarkers identified by Cox and LASSO regression were combined to explore a composite assessment approach, termed the Hematologic Marker–Patient Generated Subjective Global Assessment (HMPG-SGA), incorporating the albumin–globulin ratio (AGR). The HMPG-SGA was significantly associated with overall survival (*p* < 0.001), with an AUC of 0.616 and a C-index of 0.605. **Conclusions:** Multidimensional validation demonstrated limited prognostic discrimination of eight conventional nutritional assessment tools for overall survival in patients with hematologic malignancies. Based on existing assessment tools and integrated hematologic indicators, the HMPG-SGA was preliminarily explored as a prognostic assessment tool in hematologic malignancies.

## Simple Summary

Malnutrition is common among patients with hematologic malignancies and is closely associated with adverse clinical outcomes. Although several nutritional assessment tools are widely used in oncology, their prognostic value in hematologic cancers has not been sufficiently validated. In this large prospective cohort study including 1067 patients, we systematically evaluated eight commonly used nutritional assessment tools and found that their overall predictive ability for overall survival (OS) was limited, particularly among patients with leukemia. On this basis, we further explored whether integrating hematologic and inflammation-related biomarkers could improve prognostic assessment. By incorporating selected hematologic indicators into an optimized PG-SGA framework, we developed a modified assessment tool (HMPG-SGA), which demonstrated improved predictive performance compared with traditional tools.

## 1. Introduction

Hematologic malignancies represent a distinct clinical challenge, characterized by biological aggressiveness and therapeutic complexity, often resulting in markedly poorer survival outcomes compared to solid tumors [1,2,3,4,5]. The underlying metabolic dysregulation and chronic inflammatory milieu inherent to these malignancies lead to abnormal nutrient partitioning and promote the development of cachectic states [6,7]. This pathophysiological cascade, compounded by treatment-related toxicity, leads to irreversible functional decline, ultimately manifesting as shortened survival and impaired health-related quality of life. The unique microenvironmental disturbances in hematologic malignancies may limit the applicability of conventional nutritional assessment tools [8,9].

Contemporary nutritional assessment systems encompass multidimensional instruments: The Patient-Generated Subjective Global Assessment (PG-SGA) operationalizes dynamic nutritional monitoring through patient-reported outcomes and clinical parameters synthesis; the Global Leadership Initiative on Malnutrition (GLIM) criteria employ dual-axis diagnostic frameworks integrating phenotypic and etiological dimensions; while oncology-specific functional tools include the Karnofsky Performance Status (KPS) scale quantifying physiological reserve. Modified instruments like mPG-SGA, PG-SGA Short Form, and abPG-SGA enhance clinical feasibility through parameter optimization, whereas NRS-2002 and Scored-GLIM [10] emphasize risk stratification [10,11,12,13,14].

Although these nutritional assessment tools have demonstrated good prognostic performance in patients with solid tumors, their applicability in hematologic malignancies remains insufficiently validated [7]. This is partly attributable to fundamental biological differences between solid and hematologic cancers, including distinct tumor microenvironments, systemic inflammatory profiles, and metabolic dysregulation, which may influence the prognostic relevance of conventional nutritional indicators [5,15]. This study is the first to apply multidimensional statistical modeling and machine learning techniques, with the aim to: (1) evaluate and validate the prognostic performance of eight major assessment tools in hematologic malignancies and (2) attempt to construct a nutritional assessment method suitable for hematologic malignancies, providing a targeted framework for precision nutrition interventions.

## 2. Methods

### 2.1. Study Participants

**Figure 1:**
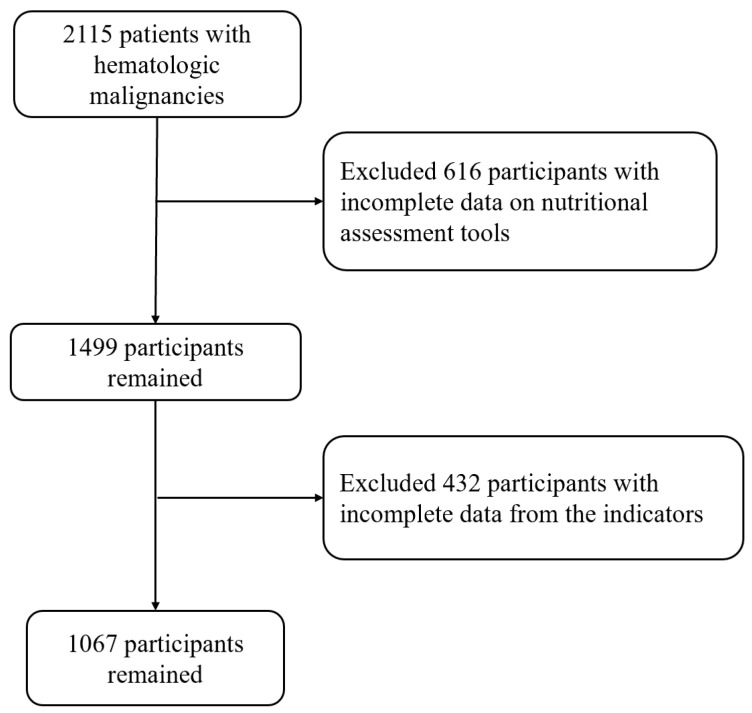
Illustrated the patient screening flowchart.

This study was conducted within the framework of the “Investigation on Nutrition Status and Clinical Outcome of Common Cancers” (INSCOC) project, registered at http://www.chictr.org.cn (Registration No. ChiCTR1800020329), which prospectively collected data from multiple Chinese institutions. Among 2115 patients with hematologic malignancies treated between 2013 and 2021, rigorous exclusion criteria were applied to ensure methodological integrity: (1) participants with incomplete clinical records, (2) those lacking follow-up data, and (3) individuals aged < 18 years. Ultimately, 1067 eligible participants were included for subsequent analysis. The study adhered to the principles of the Declaration of Helsinki, with approval obtained from the institutional ethics committee. Written informed consent was secured from all participants (Figure 1).

### 2.2. Patient Characteristics and Outcomes

A comprehensive data acquisition protocol was employed to capture multidimensional parameters encompassing demographic profiles, clinical characteristics, laboratory biomarkers, and anthropometric measurements. The standardized data framework was constructed to include variables such as age stratification, smoking and alcohol consumption patterns, comorbidities, familial cancer history, tumor histopathological classification, tumor staging according to WHO criteria, therapeutic regimens (chemotherapy/radiotherapy), nutritional support, biochemical profiles, and body composition metrics [16]. All variables were systematically sourced from institutional electronic medical record (EMR) systems. These demographic and clinicopathological variables were collected at the point of initial enrollment in the INSCOC project [17].

Nutritional status is closely related to disease progression and clinical outcomes in patients with hematologic malignancies, with overall survival (OS) serving as the key indicator of long-term prognosis. In routine clinical practice, however, the impact of malnutrition is not limited to survival alone. Poor nutritional status may also affect treatment tolerance, the occurrence of complications, and functional status, which are reflected in short- or intermediate-term outcomes such as length of hospital stay and quality of life. Therefore, using OS as the sole endpoint may not fully capture the clinical relevance of nutritional assessment tools in this patient population. Based on this consideration, OS was selected as the primary outcome to evaluate the association between nutritional assessment tools and survival. In addition, length of hospital stay (LOS) and health-related quality of life, assessed by the EORTC QLQ-C30, were included as secondary outcomes to support the robustness of the findings from the perspectives of healthcare utilization and patient functional status.

### 2.3. Nutritional Assessment

Malnutrition is common in patients with cancer and has important clinical implications. It is closely related to cancer-associated metabolic changes, chronic inflammation, reduced food intake, and treatment-related adverse effects. Many studies have shown that nutritional status reflects not only the general health of patients but also influences treatment tolerance, the risk of complications, and clinical outcomes [17]. For these reasons, nutritional assessment has become an essential part of comprehensive cancer care.

In patients with solid tumors, nutritional assessment tools such as the PG-SGA, NRS-2002, and GLIM criteria are widely used. Their associations with overall survival, perioperative complications, treatment interruption, and quality of life have been reported across different cancer types. By integrating information on weight loss, dietary intake, inflammatory status, and functional performance, these tools help identify patients at high nutritional risk and support nutritional intervention, treatment planning, and risk stratification [18]. Therefore, in solid tumors, nutritional assessment serves not only as a screening method but also as a useful tool for prognostic evaluation and clinical management.

In contrast, hematologic malignancies differ substantially from solid tumors in both biological characteristics and clinical presentation. Patients with hematologic cancers often show more pronounced systemic inflammation, immune dysfunction, and metabolic abnormalities related to bone marrow involvement. As a result, the mechanisms and clinical manifestations of malnutrition in these patients may differ from those observed in solid tumors [15].

Although existing nutritional assessment tools are supported by strong evidence in solid tumors, their prognostic value and clinical relevance in hematologic malignancies remain insufficiently validated. Systematic evaluation of the relationships between nutritional assessment tools and multiple clinical outcomes, including survival, hospital stay, and quality of life, may help clarify the role of nutritional status in disease progression and provide a basis for refining or adapting nutritional assessment strategies for patients with hematologic malignancies.

The PG-SGA, KPS, and NRS-2002 were assessed and recorded at baseline by trained staff. Based on a literature review, we identified five classification methods for PG-SGA, all of which were examined in this study. In addition, based on baseline data, we re-evaluated the GLIM, Scored GLIM, mPG-SGA, abPG-SGA, and PG-SGA SF [18,19]. The Patient-Generated Subjective Global Assessment (PG-SGA) evaluates nutritional status through seven distinct components: Weight reduction [20] (Box 1), Food intake patterns (Box 2), Symptoms (Box 3), Activity and Functional status (Box 4), Disease (Worksheet 2), Metabolic Demand (Worksheet 3), and Physical Exam (Worksheet 4). Modified versions including mPG-SGA, PG-SGA SF, and abPG-SGA represent streamlined adaptations of the original instrument, each employing unique quantification methods and diagnostic thresholds as documented in Supplementary Table S1.

The Global Leadership Initiative on Malnutrition (GLIM) diagnostic framework incorporates two core diagnostic dimensions: pathogenic-related factors (impaired nutrient intake/absorption, systemic inflammatory states, or pathological stressors) and clinical presentation indicators (unintentional body mass decline, suboptimal body mass index, or diminished lean tissue reserves). Diagnostic confirmation necessitates concurrent fulfillment of at least one pathogenic element and one clinical manifestation indicator [11]. As all enrolled oncology patients met the etiologic criteria, the evaluation focused on the clinical presentation indicators, as detailed in Supplementary Table S2.

The Scored Global Leadership Initiative on Malnutrition (sGLIM) assessment framework was constructed through nomogram-based methodology to enhance and standardize malnutrition evaluation [10]. This quantification system encompasses four distinct assessment components: non-volitional body mass reduction (scoring range 0–10), nutritional intake patterns (0–7 points), somatic protein reserves (0–6 points), and suboptimal anthropometric indices (0–4 points). Nutritional risk stratification follows a tripartite classification protocol: adequate nutritional status (aggregate scores 0–7), intermediate nutritional depletion (8–13 points), and critical nutritional compromise (scores exceeding 13 points) (Table S3).

Given the substantial differences in disease mechanisms and metabolic features between hematologic malignancies and solid tumors, additional hematologic and inflammation-related indicators were incorporated in the preliminary exploration of nutritional assessment methods. This approach aimed to better capture disease-specific characteristics of hematologic malignancies, including bone marrow involvement, systemic inflammatory activation, and immune dysfunction, thereby improving the relevance of nutritional assessment to clinical outcomes [21].

During the variable selection stage, ten indicators were initially included: AST, ALT, Hb, lymphocyte count (L), PLT, TSF, PNI, NLR, AGR, and PAR. The inclusion of these variables was based on the following considerations. First, all indicators are routinely and systematically collected in the INSCOC program, ensuring good data completeness and practical feasibility. Second, these variables reflect overall metabolic and nutritional status from multiple dimensions, including liver function, hematologic status, inflammatory response, nutritional reserves, and body composition, and have been used in previous studies on cancer nutrition and prognosis. Third, patients with hematologic malignancies commonly exhibit inflammation activation, impaired hematopoietic function, and abnormal protein metabolism, which may jointly influence nutritional assessment and its association with clinical outcomes.

It should be noted that several composite indicators, including PNI, NLR, AGR, and PAR, were emphasized in the initial variable construction. Compared with single laboratory parameters, these composite indices integrate information from different physiological domains and may provide a more stable reflection of overall patient status. Such indicators have been widely applied in oncologic research. The Prognostic Nutritional Index (PNI) combines serum albumin levels with peripheral lymphocyte counts to reflect both nutritional reserves and immune function. It has been used for prognostic evaluation and risk stratification in various solid tumors and in some hematologic malignancies. The neutrophil-to-lymphocyte ratio (NLR) is one of the most commonly used markers of systemic inflammation and reflects the balance between inflammatory activation and immune suppression. Numerous studies have reported an association between elevated NLR and poor outcomes across different cancer types, including hematologic malignancies. The albumin-to-globulin ratio (AGR) incorporates information on nutritional status and inflammatory or immune activation and may partially reduce the influence of inflammation on serum albumin alone. The platelet-to-albumin ratio (PAR) integrates platelet activation with protein nutritional status. Platelets play an important role in cancer-related inflammation, coagulation abnormalities, and tumor–host interactions, which has led to the use of PAR as an indicator of inflammatory burden and prognostic risk in some malignancies [21,22,23,24].

Overall, these composite indicators integrate signals related to inflammation, immunity, and nutrition, providing a more comprehensive perspective on the systemic condition of cancer patients and offering a reasonable theoretical basis for further investigation in hematologic malignancies. All parameters were calculated following standardized international protocols, and detailed information is provided in Supplementary Table S4.

### 2.4. Statistical Analysis

Continuous variables were summarized as mean ± standard deviation (SD) for normally distributed data or median with interquartile range (IQR) for skewed data. Student’s t-test was used for normally distributed continuous variables, while the Mann–Whitney U test was applied to non-normally distributed data. Categorical variables were reported as frequencies (percentages) and analyzed using chi-square test or Fisher’s exact test. Univariate and multivariate Cox proportional hazards models were applied to evaluate the predictive value of various nutritional assessment tools for overall survival. The predictive performance for overall survival (OS) was evaluated using area under the curve (AUC) [25] and the concordance index (C-index) [26]. The time-dependent AUC was calculated at predefined follow-up time points, with time measured in months from diagnosis. Subgroup analyses were conducted in patients with lymphoma and leukemia. LASSO regression was employed to screen hematologic biomarkers, and predictive performance was compared after stratification using optimal cutoff values. Kaplan–Meier curves were generated to assess the prognostic value of the modified nutritional assessment tool in hematologic malignancies and its subgroups, including lymphoma and leukemia. Univariate Cox regression was conducted to compare the traditional PG-SGA with the modified tool, and their predictive accuracy was assessed using AUC analysis. Statistical significance was defined as a two-sided *p*-value < 0.05. All analyses were performed using R version 4.3.3.

## 3. Results

### 3.1. Patient Baseline Characteristics

This study analyzed complete data from 1067 patients with hematologic malignancies. The baseline characteristics of the study population are presented in Table 1 and Table S4, with Table 1 summarizing demographic data and Supplementary Table S5 detailing nutritional status classifications according to different assessment tools. The cohort included 696 lymphoma cases and 371 leukemia cases. Variables independently associated with overall survival included sex, age, history of liver disease, chronic comorbidities, anemia status, family history of cancer, and smoking history. Additional baseline characteristics are provided in Table 1 and Table S5.

### 3.2. Comparative Evaluation of Predictive Performance Across Nutritional Assessment Tools

**Figure 2:**
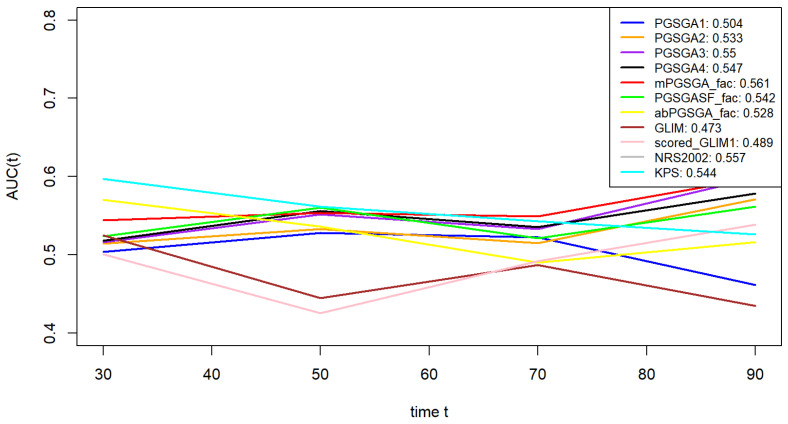
AUC values of eight nutritional assessment tools.

Multivariable analysis demonstrated significant prognostic associations among nutritional assessment tools. The Karnofsky Performance Status (KPS) [27] exhibited an inverse correlation with mortality risk (HR = 0.990 per 1-point increase, 95% CI: 0.982–0.999, *p* = 0.029), suggesting improved survival outcomes with higher functional capacity. The Patient-Generated Subjective Global Assessment (PG-SGA) [28] displayed hierarchical prognostic stratification: moderate malnutrition (4–8 points, HR = 1.408, 95% CI: 1.029–1.925, *p* = 0.032) and severe malnutrition (≥9 points, HR = 1.445, 95% CI: 1.012–2.064, *p* = 0.043) independently predicted elevated mortality. Binary stratification using PG-SGA (≤1 vs. >1) determined by predefined cutoff values further confirmed increased risk in malnourished patients (HR = 1.772, 95% CI: 1.128–2.781, *p* = 0.013). The modified PG-SGA (mPG-SGA) specifically identified severe malnutrition as a significant prognostic determinant (HR = 1.598, 95% CI: 1.129–2.262, *p* = 0.008), while moderate malnutrition showed marginal association (*p* = 0.110). Conversely, alternative classifications including PG-SGA (≤4 vs. >4) [16], PG-SGASF [29], abPG-SGA, GLIM criteria, and scored-GLIM failed to achieve statistical significance across severity strata (all *p* > 0.05). The Nutritional Risk Screening 2002 (NRS2002) approached clinical significance (HR = 1.301, 95% CI: 0.994–1.703, *p* = 0.055). Comparative evaluation indicated that PG-SGA–based stratifications, particularly higher score categories (≥9 points), showed relatively better prognostic discrimination for nutrition-associated survival outcomes in patients with hematologic malignancies (Table 2). The predictive capacity for overall survival (OS) was evaluated using time-dependent receiver operating characteristic (AUC) and concordance index (C-index) analyses. Superior performance was demonstrated by the PG-SGA three-tier classification (AUC = 0.55, C-index = 0.546) and mPG-SGA (AUC = 0.561, C-index = 0.551) compared to other tools, including NRS2002, KPS, PG-SGASF, abPG-SGA, GLIM, and sGLIM. However, none of the eight nutritional assessment tools, including PG-SGA and mPG-SGA, demonstrated satisfactory predictive accuracy for overall survival, particularly among patients with leukemia. (Table 2, Figure 2).

Significant differences in predictive performance were observed among nutritional assessment tools in hematologic malignancies through subgroup analyses. No statistically significant prognostic value was demonstrated by any nutritional assessment tools in leukemia patients (Table S6). In contrast, significant prognostic associations consistent with the overall cohort findings were observed in lymphoma patients for the PG-SGA three-tier classification (moderate/severe malnutrition: HR = 1.408/1.445) and modified mPG-SGA (severe malnutrition: HR = 1.598) (Table S7). Time-dependent receiver operating characteristic (AUC) and concordance index (C-index) evaluations indicated that all tools in leukemia subgroups showed *p*-values > 0.05, which were not considered further (Figures S1 and S2; Table S6). In lymphoma subgroups, marginally higher predictive accuracy was noted for NRS2002 (AUC = 0.602), PG-SGA three-tier classification (AUC = 0.578), and abPG-SGA (AUC = 0.578) (Figures S3 and S4; Table S7).

The comparative evaluation revealed suboptimal performance across existing nutritional assessment tools in hematologic malignancies. While the PG-SGA and mPG-SGA demonstrated relatively superior discriminative capacity for survival outcomes in the overall cohort (AUC = 0.55 and 0.561, C-index = 0.546 and 0.551), their predictive utility was markedly attenuated in leukemia subgroups. Notably, all evaluated instruments demonstrated non-significant prognostic stratification efficacy in leukemia patients, with the maximum AUC reaching only 0.544 (95% CI: 0.502–0.586). This indicates essentially random predictive performance, comparable to coin-flip accuracy. Moderate predictive accuracy was observed in lymphoma subgroups.

In the supplementary analyses, we further examined the associations between nutritional assessment tools and length of hospital stay (LOS) as well as quality of life measured by the EORTC QLQ-C30, to assess consistency with the primary overall survival (OS) findings. Overall, most tools showed patterns in LOS and QLQ-C30 that were largely consistent with those observed for OS.

Specifically, increasing scores on PG-SGA and mPG-SGA were associated with progressively longer LOS and higher QLQ-C30 scores. This indicates that higher nutritional risk identified by these tools was not only related to poorer survival, but also to prolonged hospitalization and changes in quality of life. Regarding discriminative ability, the ranking of tools by C-index for LOS and QLQ-C30 closely mirrored that observed in the OS analysis. For LOS, the C-index values of PGSGA3, PGSGA4, and PG-SGA SF were approximately 0.56, while that of mPG-SGA was around 0.55. For QLQ-C30, the C-index values of these PG-SGA–based tools increased to approximately 0.65, with mPG-SGA reaching about 0.63, all exceeding those of GLIM and NRS-2002.

Overall, similar patterns were observed across OS, LOS, and QLQ-C30, supporting the robustness of the findings. Detailed results are shown in Figures S33–S36.

Cox regression analysis was performed to evaluate components of nutritional assessment tools (Table S7). Significant prognostic predictors were identified, including PG-SGA symptom assessment (HR = 1.068, 95% CI: 1.001–1.131), disease status (HR = 2.054, 95% CI: 1.551–2.721), and GLIM handgrip strength (HGS, HR = 1.424, 95% CI: 1.175–1.725), while food intake showed borderline significance (HR = 1.124, 95% CI: 0.999–1.264). Four potential biomarkers were selected from ten blood parameters [30] using LASSO regression (Figure S37), and three were confirmed as independent predictors by further Cox analysis: neutrophil-to-lymphocyte ratio (NLR, HR = 1.380, 95% CI: 1.055–1.806), platelet-to-albumin ratio (PAR, HR = 1.380, 95% CI: 1.056–1.804), and albumin-to-globulin ratio (AGR, HR = 1.528, 95% CI: 1.170–1.996). A set of modified assessment tools was constructed by integrating these predictors. (Table S8) PG-SGA-B1 demonstrated the favorable predictive performance among the modified models [31], with significant survival discrimination shown in Kaplan–Meier curves (log-rank *p* < 0.001; Figures S5–S28). The predictive accuracy of PG-SGA-B1 (AUC = 0.616, C-Index = 0.605) was significantly higher than that of the original PG-SGA (AUC = 0.547, C-Index = 0.542; Figures S29–S32). In this study, PG-SGA-B1 was designated as HMPG-SGA.

## 4. Discussion

### 4.1. Principal Findings

Hematologic malignancies exhibit biological features that differ markedly from those of solid tumors, posing specific challenges for nutritional assessment and its interpretation in relation to prognosis. Unlike solid tumors, where nutritional deterioration is often driven by the local tumor burden or organ compression, hematologic malignancies are systemic diseases. Their progression is closely associated with disruption of the bone marrow microenvironment, immune dysregulation, and persistent systemic inflammation.

During early stages of disease, patients with hematologic malignancies may not present with obvious weight loss or reduced food intake, despite the presence of substantial metabolic alterations. Sustained activation of inflammatory cytokines, such as interleukin-6 and tumor necrosis factor-α, can promote protein catabolism, suppress albumin synthesis, and accelerate muscle loss [1,32]. These changes are not always reflected by body weight or body composition. In parallel, bone marrow involvement may lead to impaired hematopoiesis and abnormal immune cell profiles, further increasing metabolic burden and reducing nutritional efficiency. Consequently, patients may develop a state of subclinical or occult malnutrition that is difficult to identify using conventional assessment tools.

In addition, hematologic malignancies—particularly leukemia—are often characterized by rapid disease progression, intensive treatment, and highly variable short-term outcomes. Under such conditions, survival is more strongly influenced by disease aggressiveness, treatment-related toxicity, and infectious complications, which may weaken the direct association between traditional nutritional indicators and prognosis. This may partly explain the limited discriminative performance of most nutritional assessment tools observed in leukemia patients in the present study. By contrast, lymphoma patients share certain similarities with solid tumors in disease course, metabolic characteristics, and treatment strategies, which may account for the relatively better predictive performance retained by some tools in this subgroup.

Most commonly used nutritional assessment tools were originally developed in solid tumor populations or general hospitalized patients and primarily emphasize weight loss, reduced dietary intake, and subjective symptoms. In patients with hematologic malignancies, however, nutritional and metabolic disturbances are more strongly driven by systemic inflammation, immune dysfunction, and bone marrow suppression. As a result, assessment tools relying mainly on body weight and symptom reporting may underestimate true nutritional risk in this population.

Taken together, the present findings do not diminish the clinical importance of nutritional status in hematologic malignancies. Rather, they suggest that conventional nutritional assessment frameworks may not adequately capture disease-specific nutritional and metabolic risks, supporting the need to explore assessment strategies incorporating hematologic and inflammation-related indicators. In this context, the inclusion of hematologic indicators is biologically justified. In hematologic malignancies, laboratory parameters directly reflect disease activity, inflammatory burden, and immune dysfunction, all of which are closely linked to metabolic alterations and clinical outcomes. Unlike body weight or dietary intake, hematologic markers are less influenced by short-term subjective factors and may capture disease-driven nutritional deterioration that is otherwise clinically inapparent.

Moreover, composite indices may be particularly informative in this setting. By integrating multiple physiological dimensions—such as inflammation, immune status, and protein metabolism—composite indicators reduce the limitations of single laboratory parameters that are susceptible to transient fluctuations or non-specific influences. This integrative property is especially relevant in hematologic malignancies, where nutritional risk often arises from systemic inflammatory activation, bone marrow suppression, and immune imbalance rather than from isolated abnormalities. Therefore, composite hematologic indices may provide a more stable and comprehensive reflection of underlying nutritional and metabolic risk in this patient population.

To address this gap, we preliminarily developed a modified nutritional assessment approach. Components from existing tools were first screened using Cox regression to identify variables associated with survival outcomes. Hematologic-specific indicators were then incorporated to reflect disease-related nutritional deterioration. Although this composite tool demonstrated improved prognostic performance compared with individual indices, it should be regarded as an initial methodological exploration. Further validation and refinement are required, particularly in heterogeneous subgroups such as leukemia patients.

### 4.2. Strengths

The strengths of this study are as follows: It was designed to systematically evaluate the prognostic value of existing large-scale nutritional assessment tools in patients with hematologic malignancies, representing the most comprehensive analysis to date with the largest number of tools compared (*n* = 8) and the largest sample size (*n* = 1067) [9,33,34]. This study is the first to demonstrate through multidimensional statistical methods that the predictive performance of current nutritional tools for survival outcomes in hematologic malignancies is suboptimal, particularly in leukemia subgroups, providing critical evidence for developing disease-specific tools. By integrating traditional nutritional parameters with hematologic-specific biomarkers, a modified assessment framework was preliminarily established, addressing a methodological gap in this field.

### 4.3. Limitations

Several limitations of this study should be noted. The evaluation methods were limited to Cox regression, AUC, and C-index, and other statistical approaches were not incorporated, potentially restricting the comprehensiveness of the analysis. The absence of TNM staging data prevented the exploration of nutritional tools’ performance across staging subgroups, which may reduce the clinical applicability of the findings. The high proportion of subjective components in tools such as PG-SGA introduced potential bias, as these factors were not rigorously controlled. Although the newly developed tool showed improved AUC compared to existing indices, its sensitivity and specificity remained below the threshold required for direct clinical application, necessitating further refinement and validation.

The modified HMPG-SGA was constructed and tested in the same dataset, which may introduce overfitting and limit its generalizability. Although penalized regression was used, formal external validation was not performed. Accordingly, this tool should be regarded as an exploratory index, and its predictive performance requires independent validation.

## 5. Conclusions

In this study, the predictive ability of eight nutritional assessment tools in patients with hematologic malignancies was systematically evaluated. It was found that the overall predictive performance of these tools did not reach an optimal level, with significant limitations observed in the leukemia subgroup. Through the selection of key components and the integration of hematologic indicators, a novel assessment tool, HMPG-SGA, was successfully developed. Its predictive ability was significantly improved compared to traditional tools, with an increase of approximately 6.7% in AUC and 7.2% in the C-index relative to PG-SGA.

## Figures and Tables

**Table 1:** Baseline Characteristics of Patients (ALL vs. Lymphoma vs. Leukemia).

		ALL	Lymphoma	Leukemia	*p*-Value
		N = 1067	N = 696	N = 371	
Sex, N (%)	men	625 (58.6)	428 (61.5)	197 (53.1)	0.01
	women	442 (41.4)	268 (38.5)	174 (46.9)	
age	<65	684 (64.1)	408 (58.6)	276 (74.4)	<0.001
	≥65	383 (35.9)	288 (41.4)	95 (25.6)	
liver (%)	No	1026 (96.2)	662 (95.1)	364 (98.1)	0.024
	Yes	41 (3.8)	34 (4.9)	7 (1.9)	
Chronic disease (%)	No	894 (83.8)	570 (81.9)	324 (87.3)	0.027
	Yes	173 (16.2)	126 (18.1)	47 (12.7)	
anemia (%)	No	1026 (95.6)	674 (96.8)	346 (93.3)	0.011
	Yes	47 (4.4)	22 (3.2)	25 (6.7)	
Family history of cancer (%)	No	992 (93)	633 (90.9)	359 (96.8)	<0.001
	Yes	75 (7)	63 (9.1)	12 (3.2)	
Smoking (%)	No	710 (66.5)	430 (61.8)	280 (75.5)	<0.001
	Yes	357 (33.5)	266 (38.2)	91 (24.5)	
TNM (%)	0	518 (48.5)	206 (29.6)	312 (84.1)	<0.001
	1	7 (4.4)	46 (6.6)	1 (0.3)	
	2	95 (8.9)	93 (13.4)	2 (0.5)	
	3	106 (9.9)	101 (14.5)	5 (1.3)	
	4	171 (16)	163 (23.4)	8 (2.2)	
	5	130 (12.2)	87 (12.5)	43 (11.6)	
chemotherapy (%)	No	224 (21)	178 (25.6)	46 (12.4)	<0.001
	Yes	843 (79)	518 (74.4)	325 (87.6)	
radiotherapy (%)	No	1017 (95.3)	653 (93.8)	364 (98.1)	0.003
	Yes	50 (4.7)	43 (6.2)	7 (1.9)	

**Table 2:** Univariate Survival Analysis of Eight Nutritional Assessment Tools.

		N	HR (95%CI)	*p*-Value
KPS			0.990 [0.982, 0.999]	0.029
PG-SGA Three-Classification				
PGSGA (0~3)	Well nourished	406		
PGSGA (4~8)	Moderately	402	1.408 [1.029, 1.925] *	0.032
PGSGA (≥9)	severely	259	1.445 [1.012, 2.064] *	0.043
PG-SGA Two-Classification1				
PGSGA (≤1)	Well nourished	148		
PGSGA (>1)	Malnourished	919	1.772 [1.128, 2.781] *	0.013
PG-SGA Two-Classification2				
PGSGA (≤4)	Well nourished	480		
PGSGA (>4)	Malnourished	587	1.249 [0.951, 1.640]	0.110
PG-SGA Four-Classification				
PGSGA4 (0~1)	Well nourished	148		
PGSGA4 (2~3)	Mildly nourished	258	1.525 [0.910, 2.559]	0.109
PGSGA4 (4~8)	Moderately nourished	402	1.845 [1.149, 2.965] *	0.011
PGSGA4 (≥9)	Severely nourished	259	1.894 [1.145, 3.133] *	0.013
mPG-SGA	Well nourished	287		
	Moderately	331	1.353 [0.934, 1.961]	0.110
	Severely	449	1.598 [1.129, 2.262] **	0.008
PG-SGA SF	Well nourished	373		
	Moderately	373	1.223 [0.883, 1.695]	0.225
	Severely	321	1.371 [0.977, 1.925]	0.068
abPG-SGA	Well nourished	695		
	Malnourished	372	1.139 [0.863, 1.503]	0.356
GLIM	Well nourished	399		
	Moderately	526	1.186 [0.866, 1.625]	0.287
	Severely	142	1.150 [0.742, 1.784]	0.531
Scored-GLIM	Well nourished	399		
	Moderately	173	1.342 [0.902, 1.996]	0.147
	Severely	495	1.127 [0.818, 1.552]	0.465
NRS-2002	Without nutritional risk	539		
	At nutritional risk	582	1.301 [0.994, 1.703]	0.055

* *p* < 0.05; ** *p* < 0.01.

## Data Availability

The datasets used and/or analyzed during the current study are available from the corresponding author on reasonable request.

## Supplementary Materials

The following supporting information can be downloaded at: https://www.mdpi.com/article/10.3390/cancers18050765/s1. Table S1: Scoring criteria of PG-SGA, mPG-SGA, PG-SGA SF, and abPG-SGA; Table S2: Scoring criteria of GLIM; Table S2: Scoring criteria of Scored GLIM; Table S4: Hematologic indicators; Table S5: Nutrition Assessment Tool-ALL-COX; Table S6: Nutrition Assessment Tool- Leukemia-COX; Table S7: Nutrition Assessment Tool- Lymphoma –COX; Table S8: New nutritional tool; Figure S1: Leukemia-AUC; Figure S2: Leukemia-C-Index; Figure S3: Lymphoma –AUC; Figure S4: Lymphoma-C-Index; Figure S5: PGSGA_A; Figure S6: PGSGA_A1; Figure S7: PGSGA_A3; Figure S8: PGSGA_A4; Figure S9: PGSGA_B; Figure S10: PGSGA_B1 (HMPGSGA); Figure S11: PGSGA_B3; Figure S12: PGSGA_B4; Figure S13: PGSGA_C; Figure S14: PGSGA_C1; Figure S15: PGSGA_C3; Figure S16 PGSGA_C4; Figure S17: PGSGA_A (Two Classification); Figure S18: PGSGA_A1 (Two Classification); Figure S19: PGSGA_A3 (Two Classification); Figure S20: PGSGA_A4 (Two Classification); Figure S21: PGSGA_B (Two Classification); Figure S22: PGSGA_B1 (HMPGSGA) (Two Classification); Figure S23: PGSGA_B3 (Two Classification); Figure S24: PGSGA_B4 (Two Classification); Figure S25: PGSGA_C (Two Classification); Figure S26: PGSGA_C1 (Two Classification); Figure S27: PGSGA_C3 (Two Classification); Figure S28: PGSGA_C4 (Two Classification); Figure S29: Binary Classification AUC; Figure S30: Binary Classification C-Index; Figure S31: Four-Class Classification AUC; Figure S32: Four-Class Classification C-Index; Figure S33: β (95%CI) of LOS and nutritional tools; Figure S34: β (95%CI) of QC30 and nutritional tools; Figure S35: C-Index of LOS with nutritional tools; Figure S36: C-Index of QC30 with nutritional tools; Figure S37: Lasso Regression: Hematological Parameters.
