# Supplementary material for: Application and Modification of Nutritional Assessment Tools in Hematologic Malignancies
Source: Cancers (Basel). 2026 Feb 27;18(5):765. doi: 10.3390/cancers18050765 (PMC12985334; doi:10.3390/cancers18050765)
Supplement: Supplementary file 1 [file cancers-18-00765-s001.zip › cancers-4117574-supplementary.pdf]

SUPPLEMENTARY MATERIAL

Table S1

	Worksheet 1				Worksheet 2(Disease)	Worksheet 3(Metabolic Demand)	Worksheet4(Physical Exam)	Classification criteria
PGSGA	Box1(Weight)	Box2(Food intake)	Box3(Symptoms)	Box4(Activities and Function)	age ≥65			**PG-SGA Three- Classification**<br>Well-nourished (0-3 points)<br>Moderate-malnutrition(4-8points)<br>Severe malnutrition (≥9 points)<br>**PG-SGA Two- Classification1**<br>Well-nourished (≤1points)<br>Malnutrished (>1points)<br>**PG-SGA Two- Classification2**<br>Well-nourished (≤4points)<br>Malnutrished (>4points)<br>**PG-SGA Four- Classification**<br>Well-Nourished (0-1 points)<br>Mildly-Malnourished (2~3points)<br>Moderate-Malnourished(4-8points)<br>Severe Malnourished (≥9 points)
mPGSGA	Box1	Box2	Box3(Delete oral ulcers)	Box4	age ≥65	----	----	Well-nourished (0-2 points)<br>Moderate malnutrition (3-6 points)<br>Severe malnutrition (≥7 points)
PGSGASF	Box1	Box2	Box3(Add fatigue)	Box4	----	----	----	Well-nourished (0-3 points)<br>Moderate malnutrition (4-8 points)<br>Severe malnutrition (≥9 points)
abPGSGA	Box1	Box2	Box3(Add fatigue and delete other)	Box4	----	----	----	Malnutrition(≥6 points)

Scoring criteria of PG-SGA, mPG-SGA, PG-SGA SF, and abPG-SGA

Table S2 GLIM

Grade	Phenotypical criteria<br>Weight loss (%)	Low BMI (kg/m2)	Reduced muscle mass
Moderate malnutrition(I)	5-10% within the past 6 months, or 10-20% beyond 6 months	<18.5 if < 70 year, <20 if ≥70 year	CC< 29 if female， CC<30 if male<br>HGS/W < 0.2144 if female,<br>HGS/W < 0.3305 if male<br>MAMC < 17.06 if female,<br>MAMC <18.66 if male
Severe malnutrition(II)	>10% within the past 6 months, or >20% beyond 6 months	Not applicable, no Asian standards	CC< 27 if female， CC<27.5 if male<br>HGS/W < 0.1375 if female,<br>HGS/W < 0.2267 if male<br>MAMC < 15.08 if female,<br>MAMC <16.49 if male

Scoring criteria of GLIM

Table S3 Scored GLIM

GLIM	sorts	Scored-GLIM Points	Scored-GLIM Grade
GLIM I	Weight loss	6	Well-Nourished (0-2.7points)
	Low BMI	7	Mildly-Malnourished (2.7~7points)
	Reduced muscle mass	3	Moderate-Malnourished (8.1-12.8points)
	disease	7	Severe Malnourished (13.8-26.8 points)
GLIM II	Weight loss	10	
	Low BMI	Not applicable	
	Reduced muscle mass	6	
	disease	7	

Scoring criteria of Scored GLIM

Table S4 Hematologic indicators

	PNI	NLR	AGR	ALI	PAR
Cut-off	56.65	3	1.34	24.68	4.64

Table S5 Nutrition Assessment Tool-ALL-COX

	HR (95%CI)	P-value
**PG-SGA**		
Weight	1.031[0.974,1.091	0.297
Intake	1.124[0.999,1.264]	0.052
Symptom	1.068[1.008,1.131] *	0.026
Disease	1.975[1.469,2.656] ***	<0.001
Activities and Function	1.050[0.907,1.217]	0.513
Metabolic Demand	0.948[0.790,1.139]	0.571
Physical Exam		
Absence of obesity	1.066[0.876,1.296]	0.523
Muscle	1.134[0.941,1.366]	0.186
Edema	1.274[0.740,2.194]	0.382
**mPGSGA**		
Weight	1.031[0.974,1.091	0.297
Intake	1.124[0.999,1.264]	0.052
Symptom Delete oral ulcers	1.071[1.010,1.135] *	0.022
Activities and Function	1.050[0.907,1.217]	0.513
**PGSGASF**		
Weight	1.031[0.974,1.091	0.297
Intake	1.124[0.999,1.264]	0.052
Symptom and Fatigue	1.070[1.016,1.126] *	0.011
Activities and Function	1.050[0.907,1.217]	0.513
**abPGSGA**		
Weight	1.031[0.974,1.091	0.297
Intake	1.124[0.999,1.264]	0.052
Fatigue	1.229[1.007,1.501] *	0.043
Activities and Function	1.050[0.907,1.217]	0.513
**GLIM**		
CC	1.120[0.914,1.372]	0.274
Weight	0.927[0.786,1.093]	0.366
HGS	1.424[1.175,1.725] ***	<0.001
MAMC	0.724[0.550,0.954] *	0.022
LBMI	1.005[0.731,1.381]	0.975
**Scored GLIM**		
Weight	0.986[0.955,1.018]	0.396
LBMI	1.001[0.956,1.047]	0.975
Muscle	1.052[0.989,1.118]	0.107

p<0.001, ***, p<0.01, **, p<0.05, *

Table S6 Nutrition Assessment Tool- Leukemia-COX

	HR（95%CI）	P-value
KPS	0.989[0.975,1.003]	0.122
**PG-SGA Three- Classification**		
Moderately (4~8)	1.278[0.765,2.134]	0.348
Severely (≥9)	1.240[0.694,2.216]	0.467
**PG-SGA Two- Classification1**		
Malnourished (>1)	1.237[0.597,2.565]	0.567
**PG-SGA Two- Classification2**		
Malnourished (>4)	1.033[0.666,1.605]	0.883
**PG-SGA Four- Classification**		
mildly nourished (2~3)	1.031[0.431,2.468]	0.945
moderately nourished (4~8)	1.304[0.612,2.778]	0.492
severely nourished (≥9)	1.265[0.567,2.823]	0.566
mPGSGA(3-6points)	1.613[0.623,4.171]	0.324
mPGSGA (>=7 points)	1.711[0.685,4.275]	0.250
PGSGASF (4-8points)	1.515[0.640,3.585]	0.345
PGSGASF (>=9 points)	1.364[0.582,3.197]	0.475
abPGSGA (>=6 points)	0.899[0.578,1.397]	0.636
GLIM I	1.323[0.776,2.255]	0.303
GLIM II	1.383[0.695,2.752]	0.356
Scored GLIM I	1.820[0.990,3.345]	0.054
Scored GLIM II	1.163[0.672,2.013]	0.588
NRS2002	0.910[0.596,1.388]	0.660

Table S7 Nutrition Assessment Tool- Lymphoma -COX

	HR (95%CI)	P-value
KPS	0.991[0.980,1.001]	0.088
**PG-SGA Three- Classification**		
Moderately (4~8)	1.657[1.102,2.493]	0.015
Severely (≥9)	1.644[1.040,2.599]	0.033
**PG-SGA Two- Classification1**		
Malnourished (>1)	2.200[1.236,3.917]	0.007
**PG-SGA Two- Classification2**		
Malnourished (>4)	1.528[1.073,2.176]	0.019
**PG-SGA Four- Classification**		
mildly nourished (2~3)	1.798[0.939,3.443]	0.077
moderately nourished (4~8)	2.412[1.306,4.453]	0.005
severely nourished (≥9)	2.403[1.254,4.605]	0.008

mPGSGA (3-6points)	1.692[0.884,3.236]	0.112
mPGSGA (>=7 points)	2.104[1.102,4.016]	0.024
PGSGASF(4-8points)	0.946[0.578,1.546]	0.823
PGSGASF (>=9 points)	1.418[0.873,2.302]	0.158
abPGSGA (>=6 points)	1.708[1.195,2.443]	0.003
GLIM（Moderately）	1.175[0.792,1.744]	0.423
GLIM（Severely）	1.125[0.628,2.015]	0.691
Scored GLIM（Moderately）	1.121[0.640,1.964]	0.689
Scored GLIM（Severely）	1.178[0.793,1.749]	0.418
NRS2002	1.800[1.266,2.557]	0.001

Table S8 New nutritional tool

	BOX1(Food Intake)	BOX2(Symptom)	BOX3(Age)	HGS	AGR	NLR	PAR
PGSGA_A	BOX1	BOX2 Add fatigue	BOX3				
PGSGA_A1	BOX1	BOX2 Add fatigue	BOX3	HGS	AGR		
PGSGA_A3	BOX1	BOX2 Add fatigue	BOX3	HGS		NLR	
PGSGA_A4	BOX1	BOX2 Add fatigue	BOX3	HGS			PAR
PGSGA_B	BOX1	Nausea、Xerostomia、Foul smell、Loss of appetite	BOX3				
PGSGA_B1	BOX1	Nausea、Xerostomia、Foul smell、Loss of appetite	BOX3	HGS	AGR		
PGSGA_B3	BOX1	Nausea、Xerostomia、Foul smell、Loss of appetite	BOX3	HGS		NLR	
PGSGA_B4	BOX1	Nausea、Xerostomia、Foul smell、Loss of appetite	BOX3	HGS			PAR
PGSGA_C		BOX2 Add fatigue	BOX3				
PGSGA_C1		BOX2 Add fatigue	BOX3	HGS	AGR		
PGSGA_C3		BOX2 Add fatigue	BOX3	HGS		NLR	
PGSGA_C4		BOX2 Add fatigue	BOX3	HGS			PAR

Scoring criteria of New Tool

HMPG-SGA=PGSGA_B1

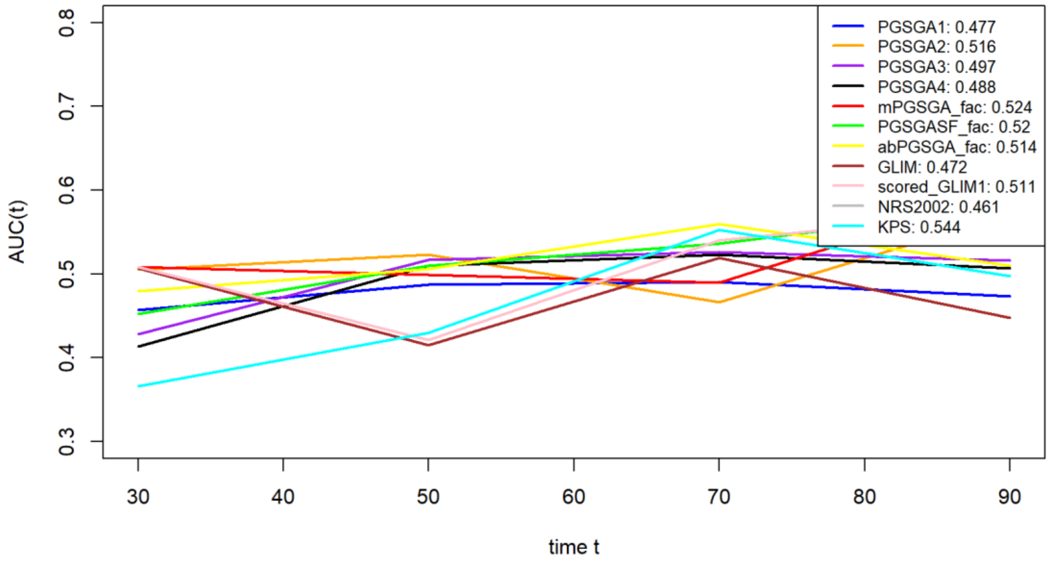

Figure S1 Leukemia-AUC

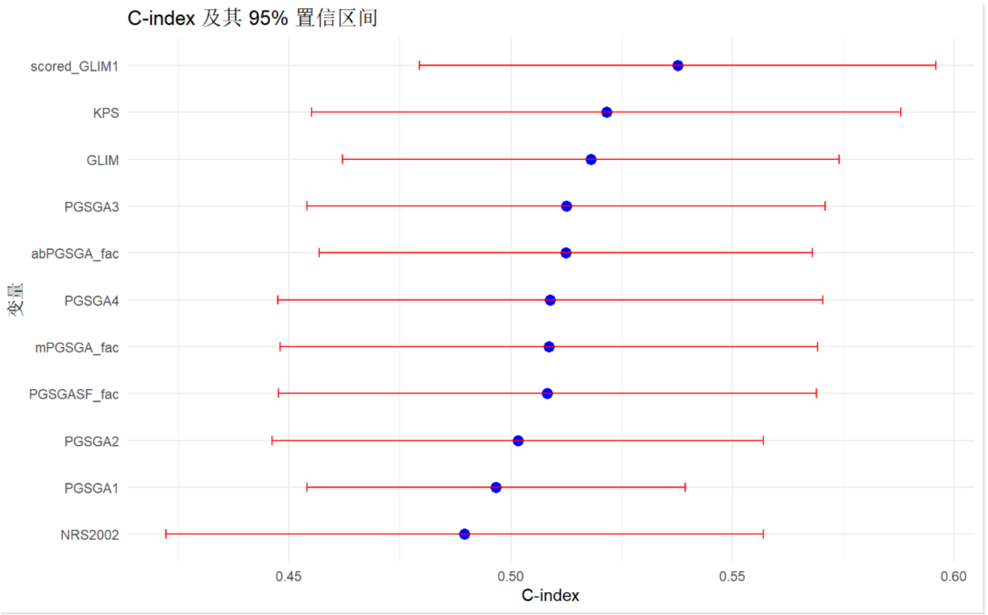

Figure S2 Leukemia-C-Index

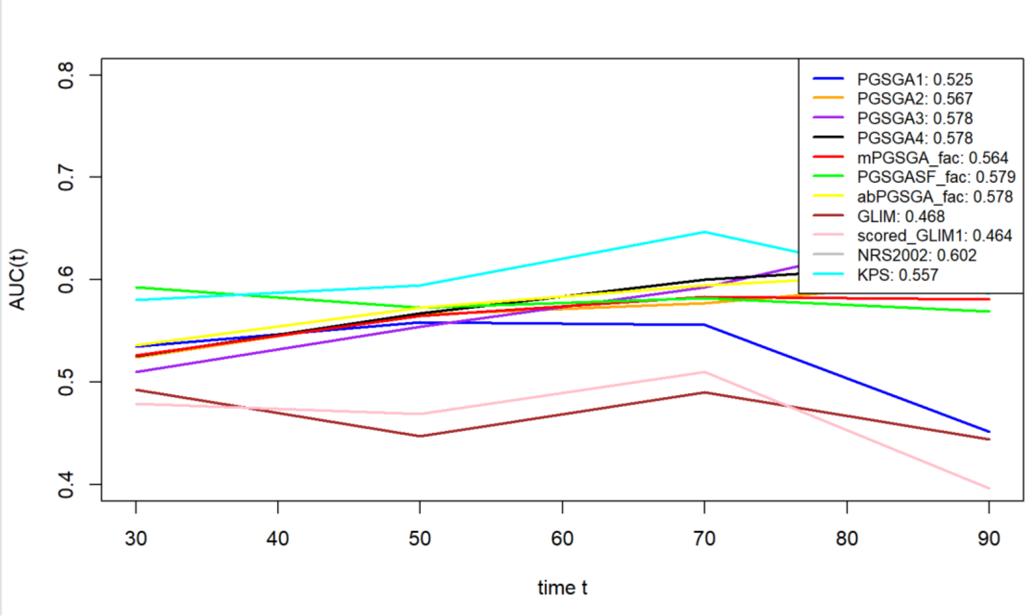

Figure S3 Lymphoma -AUC

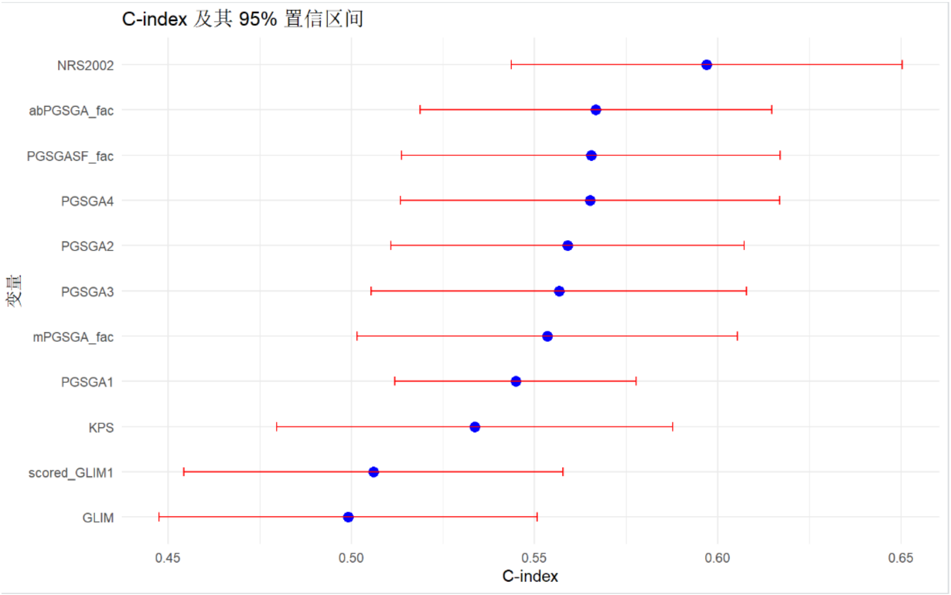

Figure S4 Lymphoma-C-Index

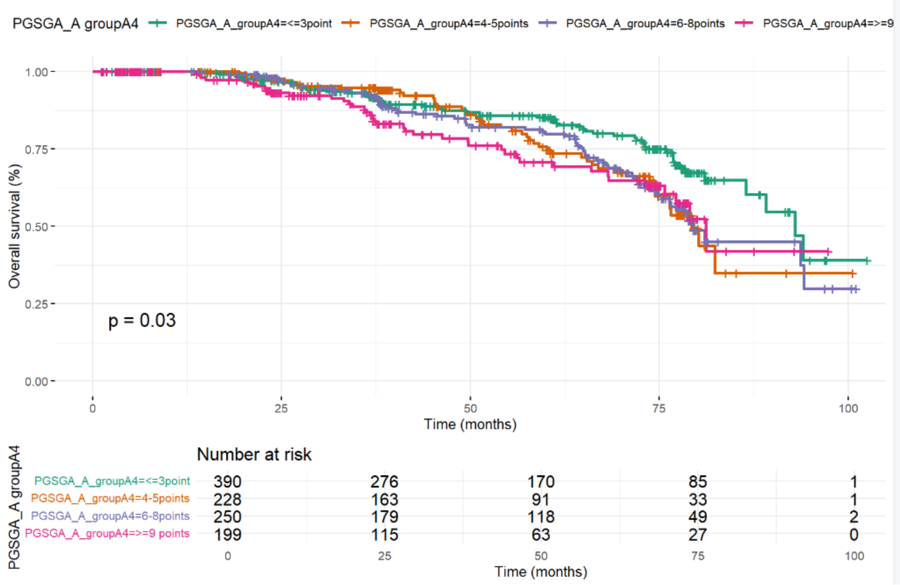

Figure S5 PGSGA_A

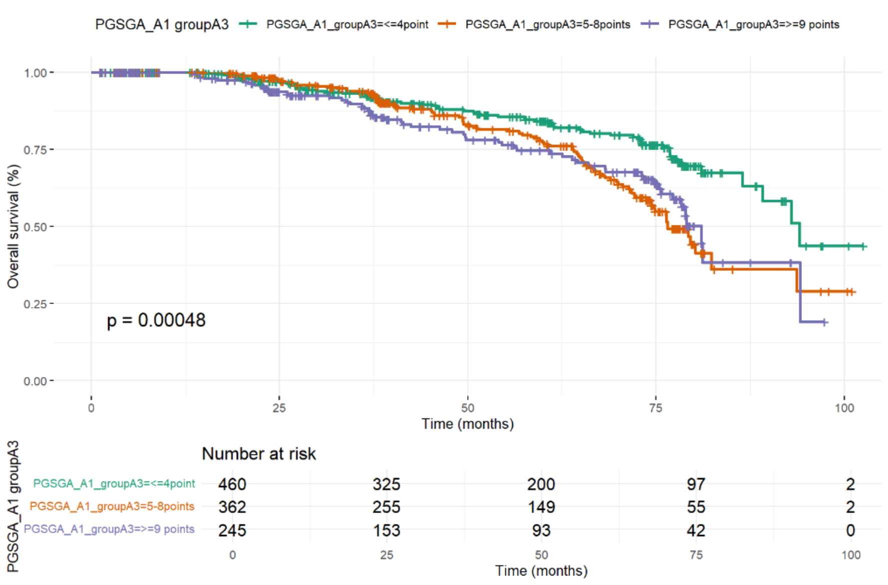

Figure S6 PGSGA_A1

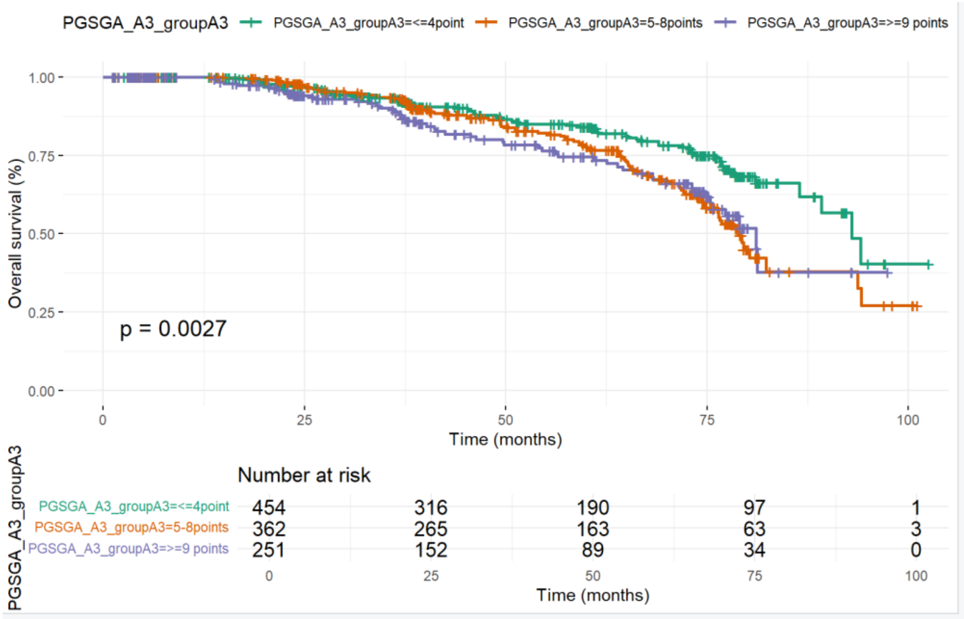

Figure S7 PGSGA_A3

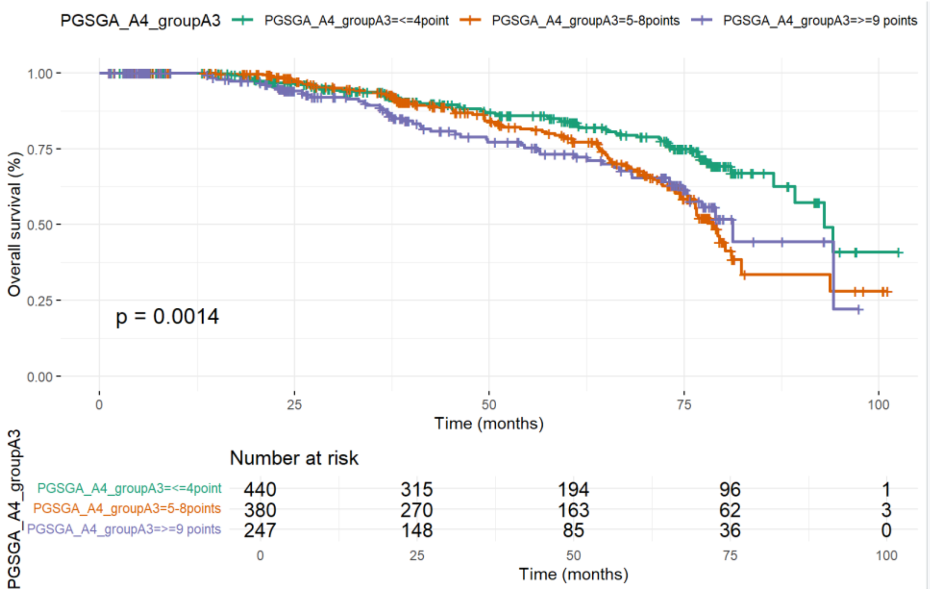

Figure S8 PGSGA_A4

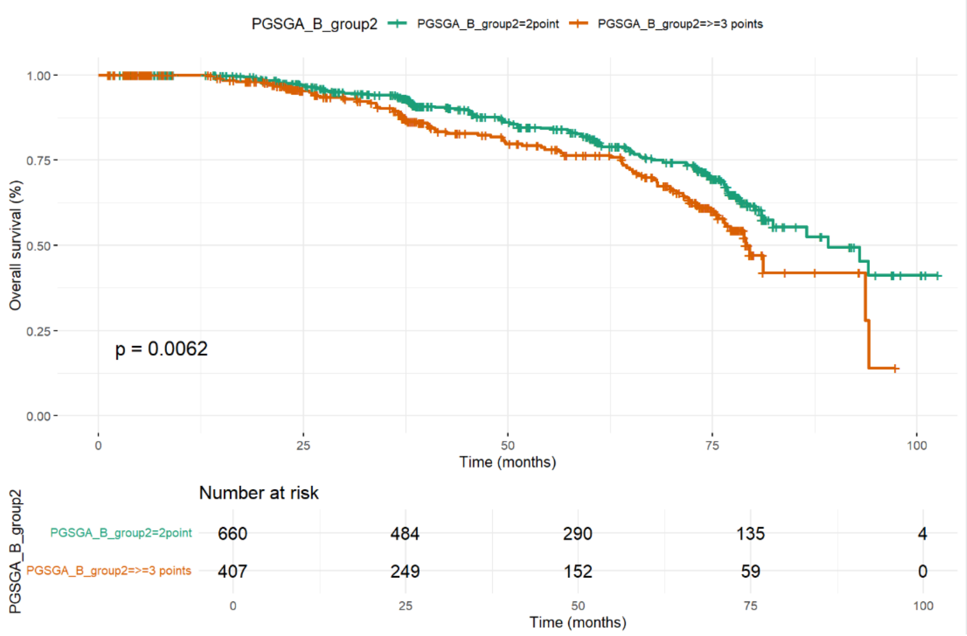

Figure S9 PGSGA_B

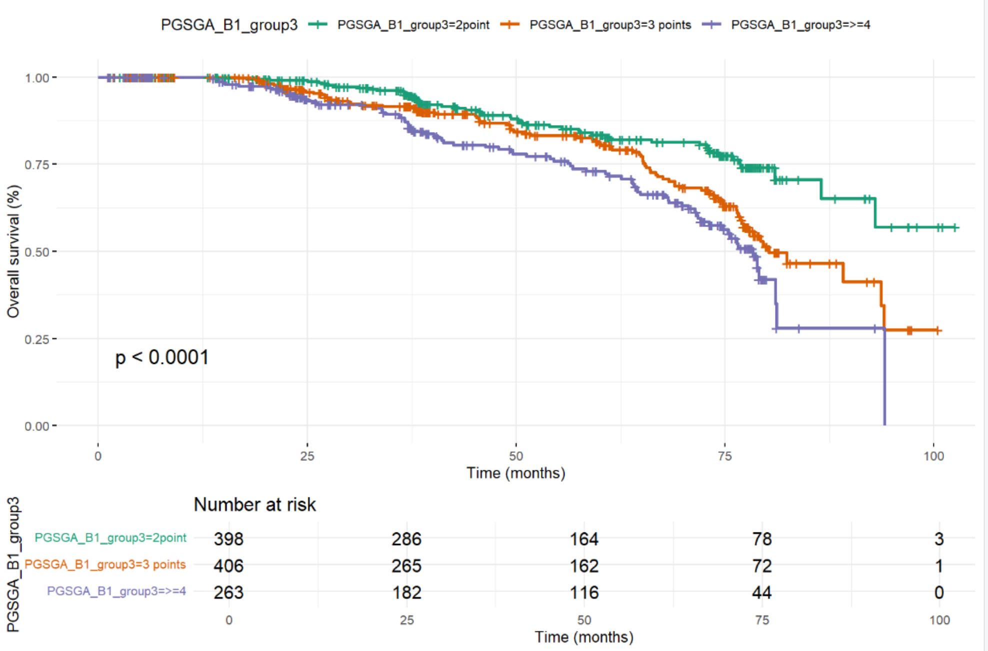

Figure S10 PGSGA_B1=HMPGSGA

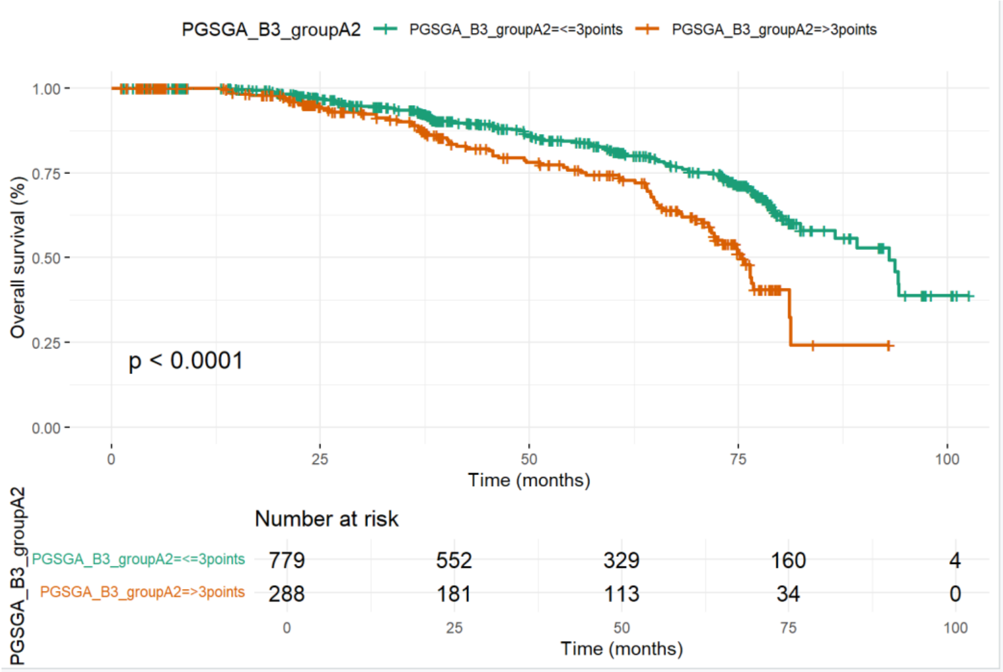

Figure S11 PGSGA_B3

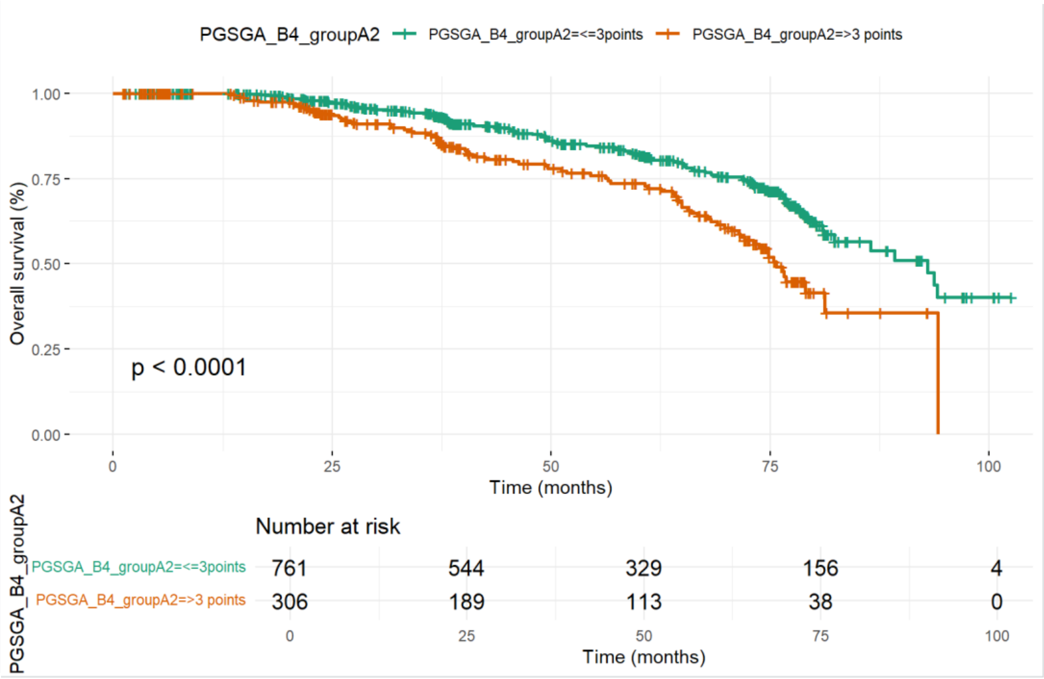

Figure S12 PGSGA_B4

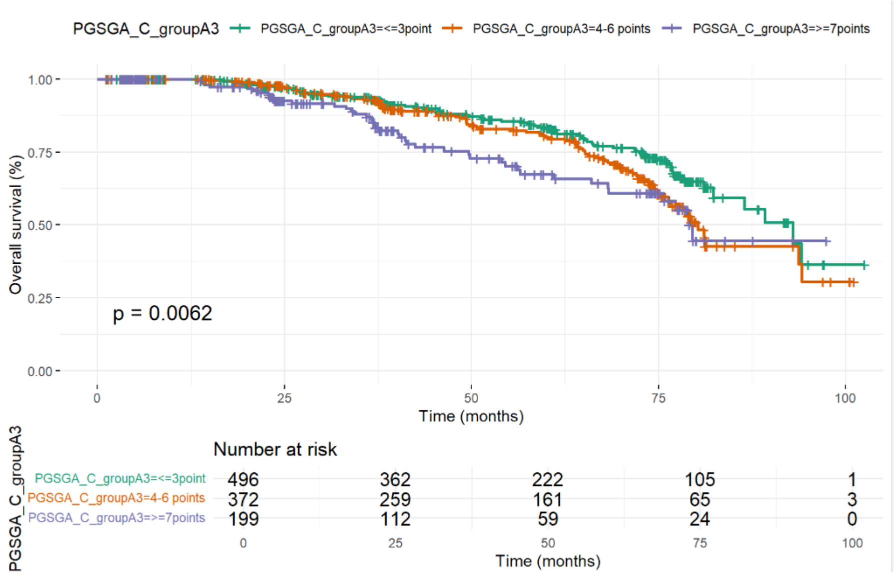

Figure S13PGSGA_C

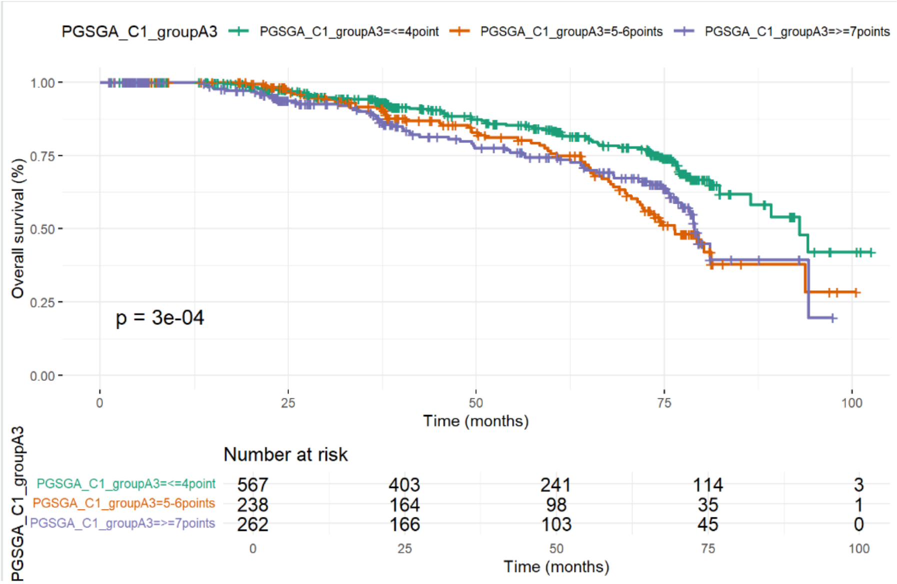

Figure S14 PGSGA_C1

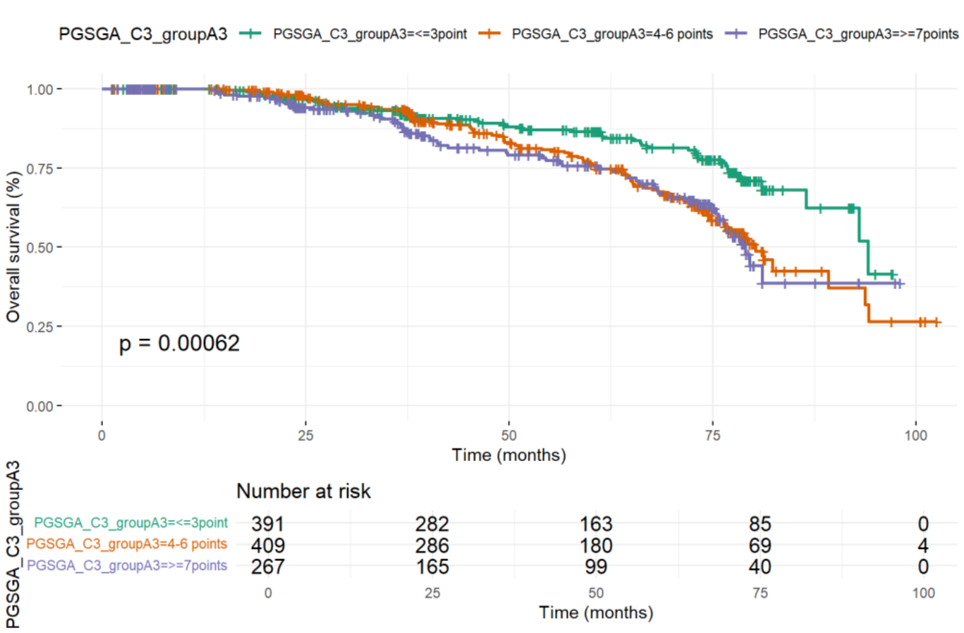

Figure S15 PGSGA_C3

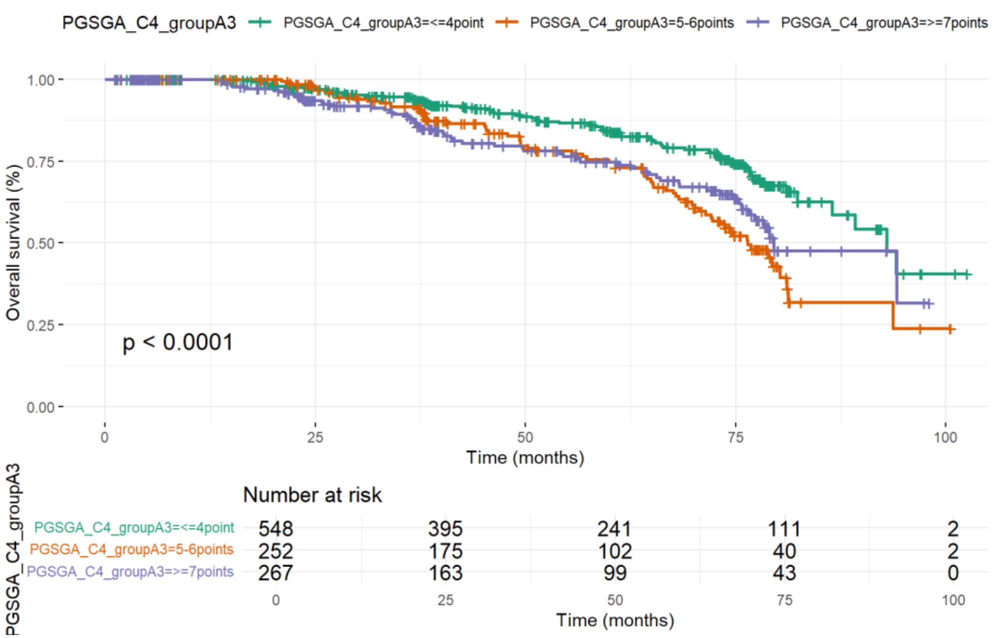

Figure S16 PGSGA_C4

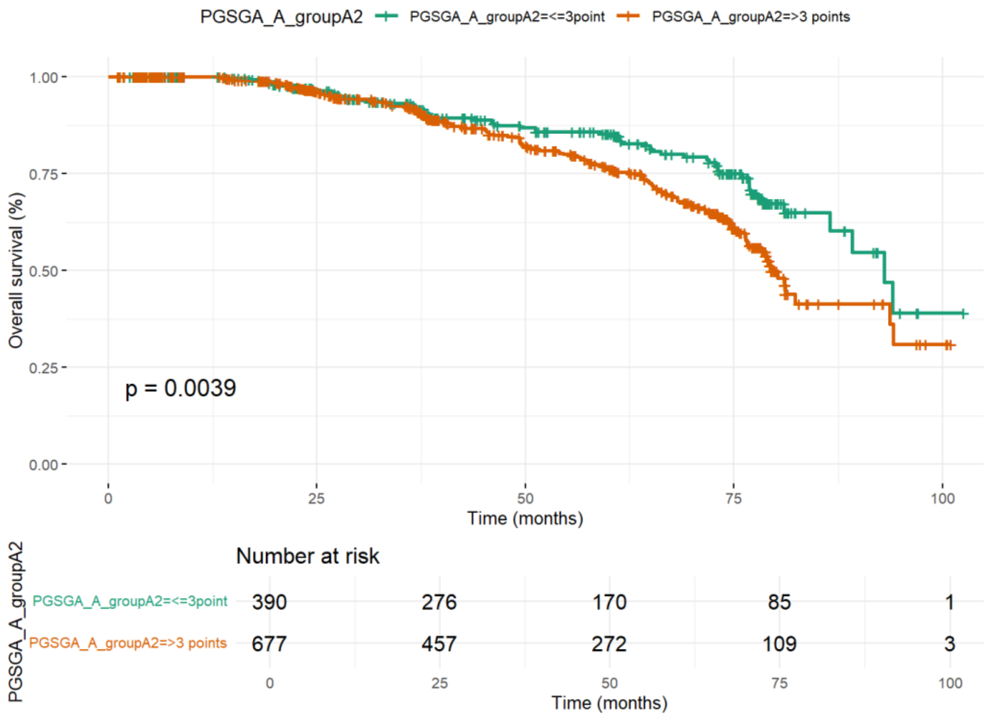

Figure S17 PGSGA_A

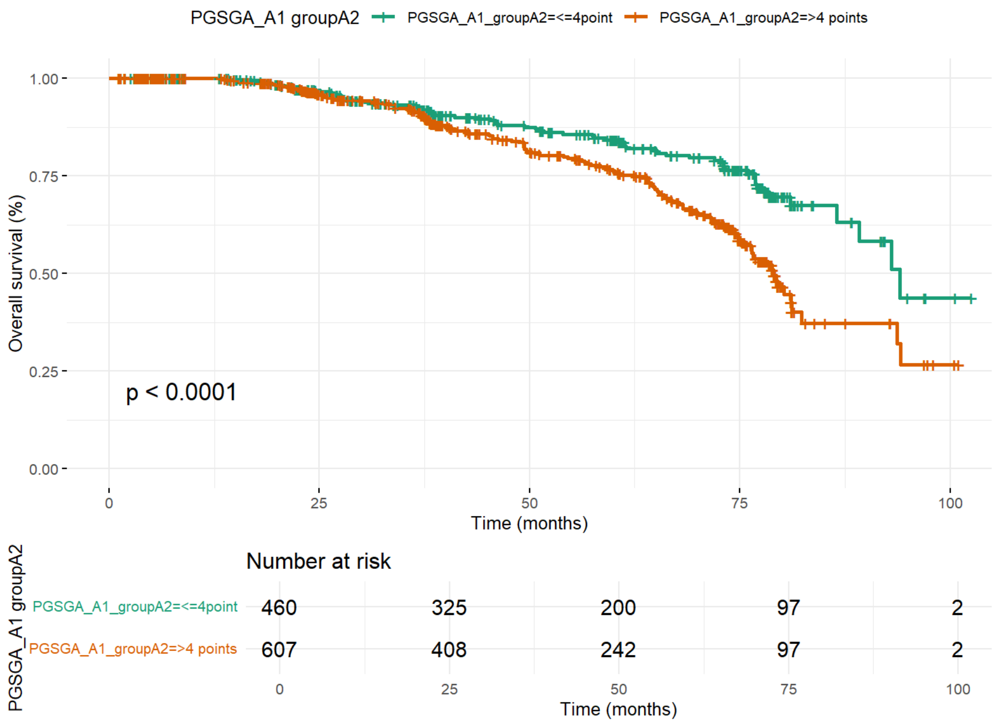

Figure S18 PGSGA_A1

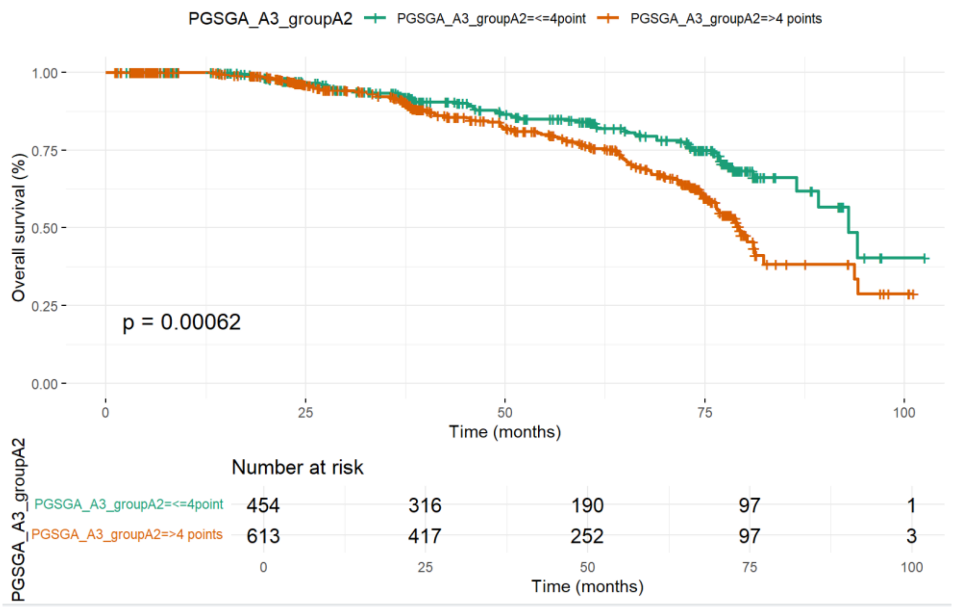

Figure S19 PGSGA_A3

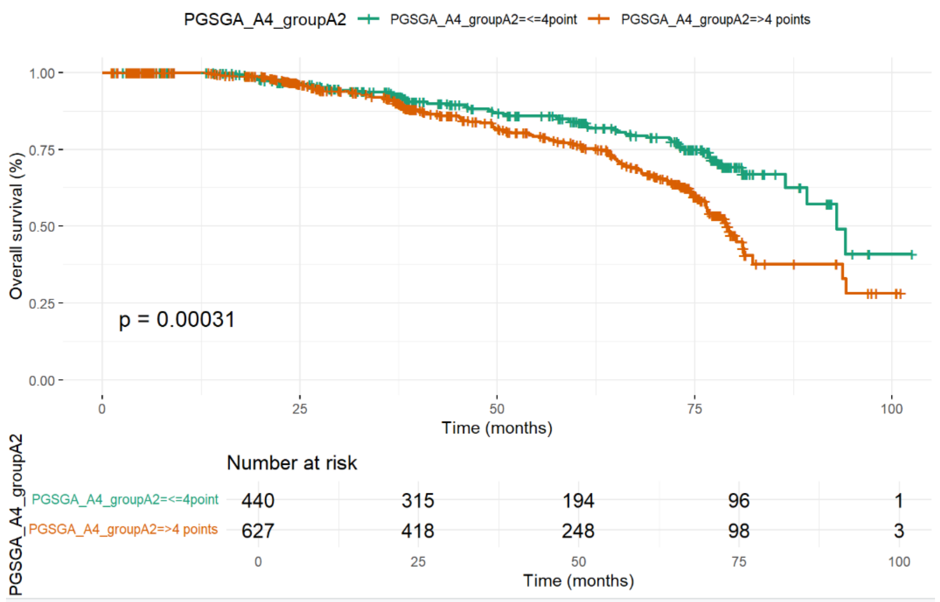

Figure S20 PGSGA_A4

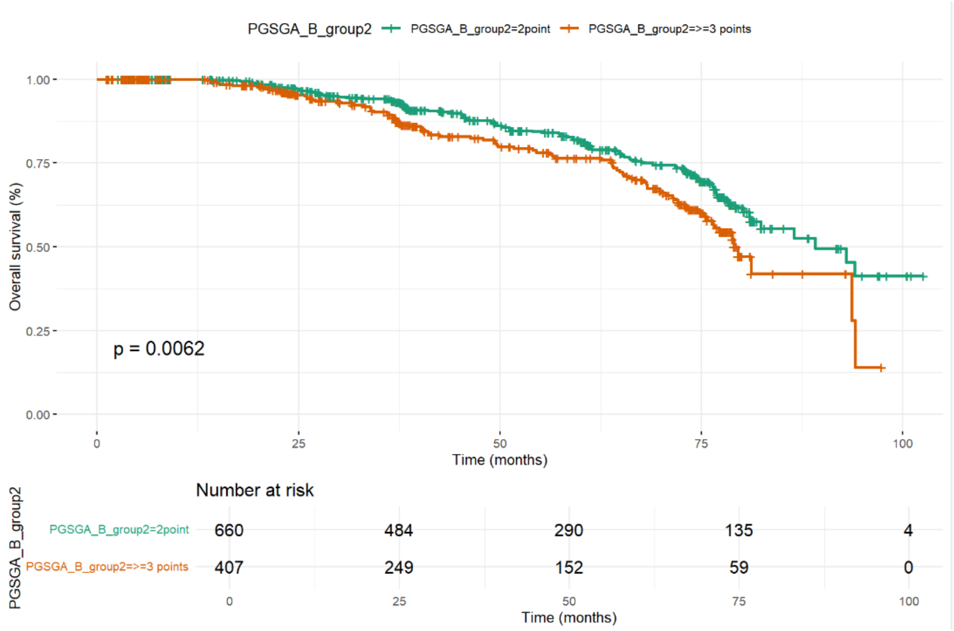

Figure S21 PGSGA_B

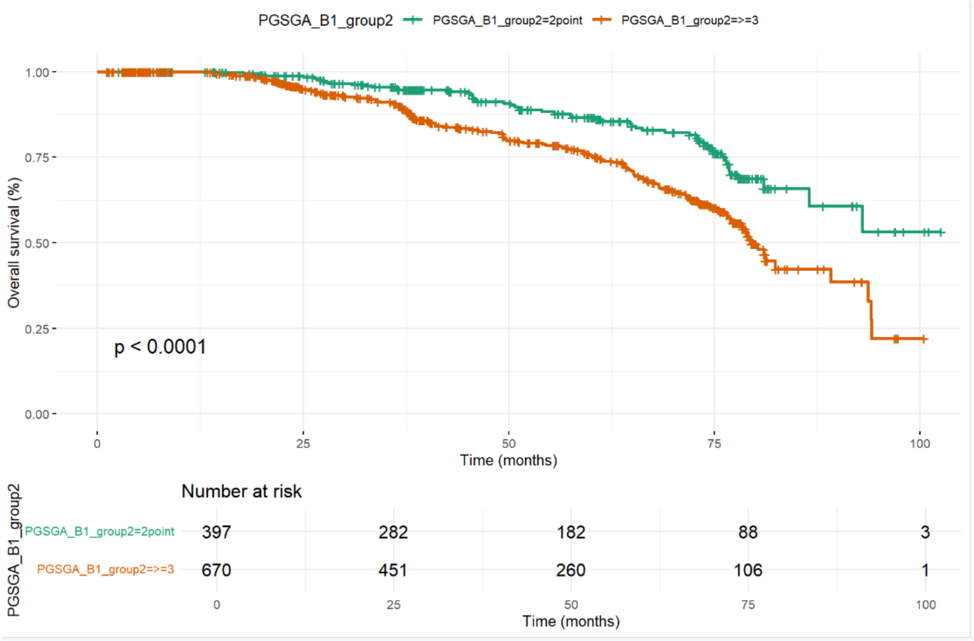

Figure S22 PGSGA_B1=HMPGSGA

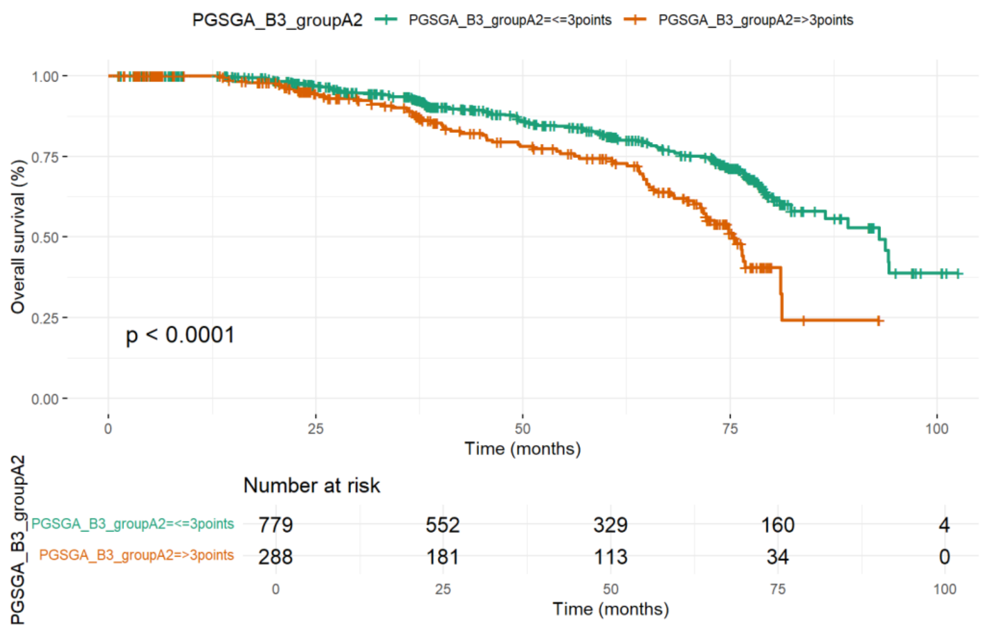

Figure S23 PGSGA_B3

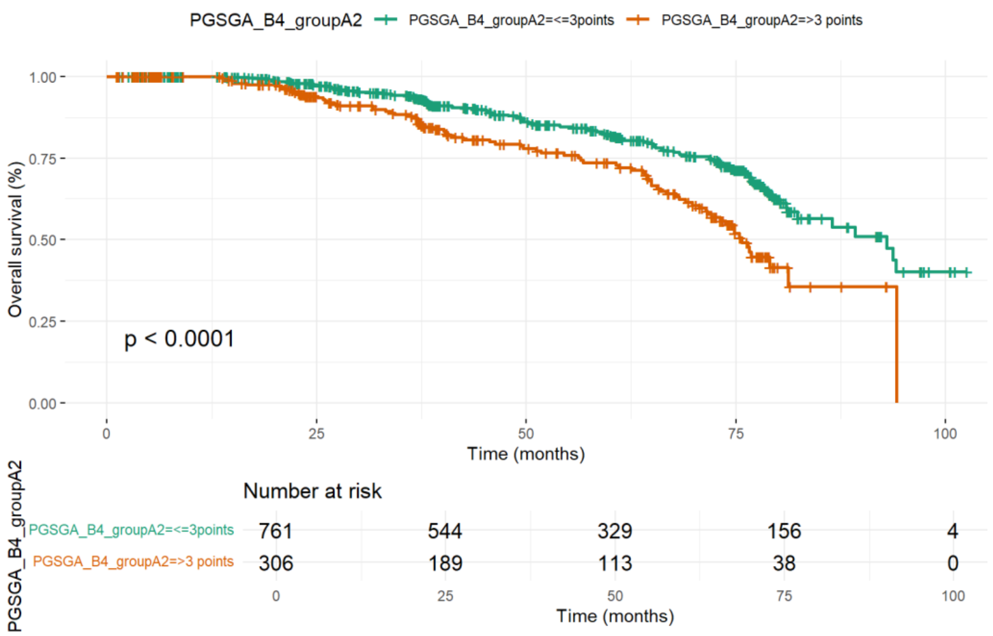

Figure S24 PGSGA_B4

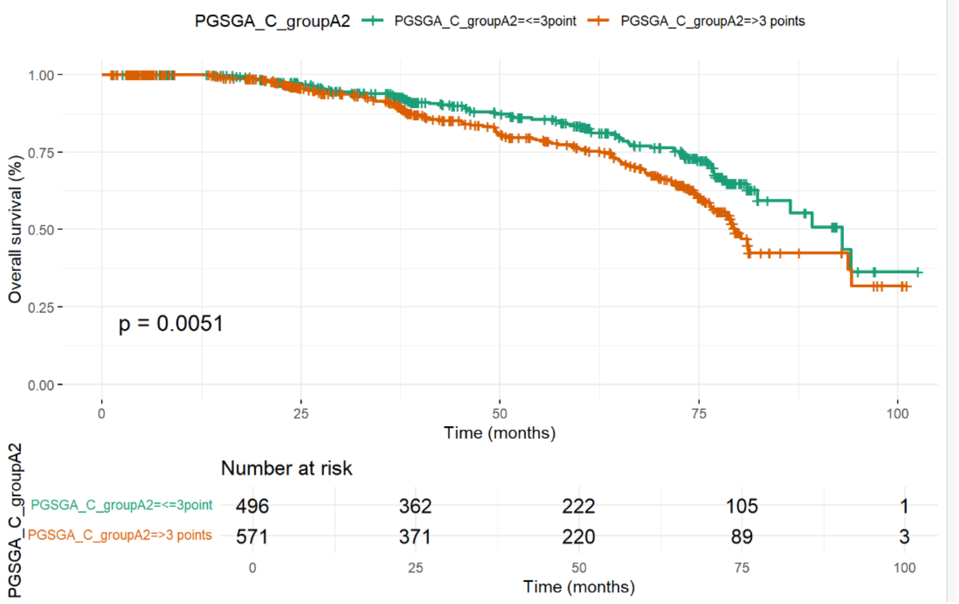

Figure S25 PGSGA_C

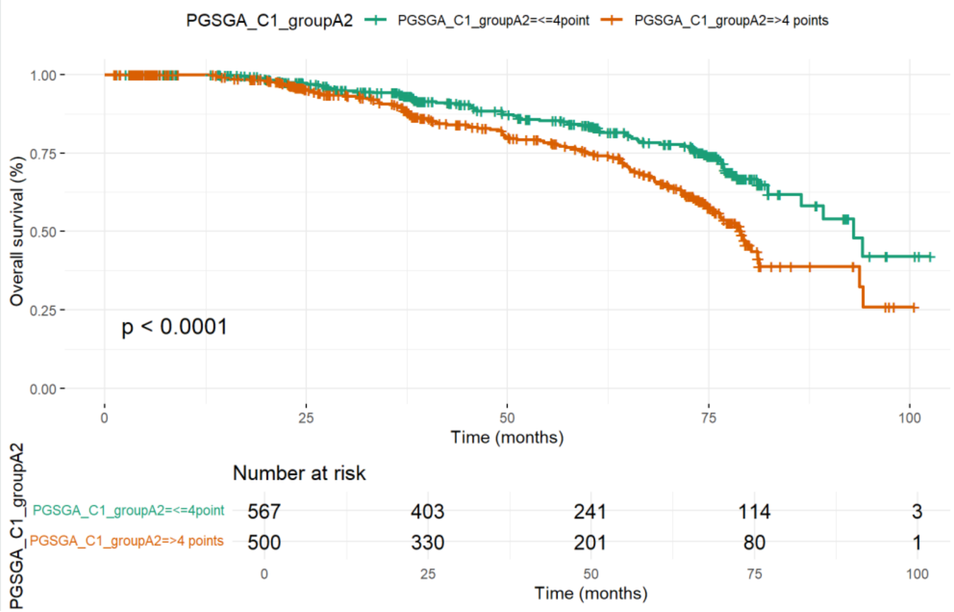

Figure S26 PGSGA_C1

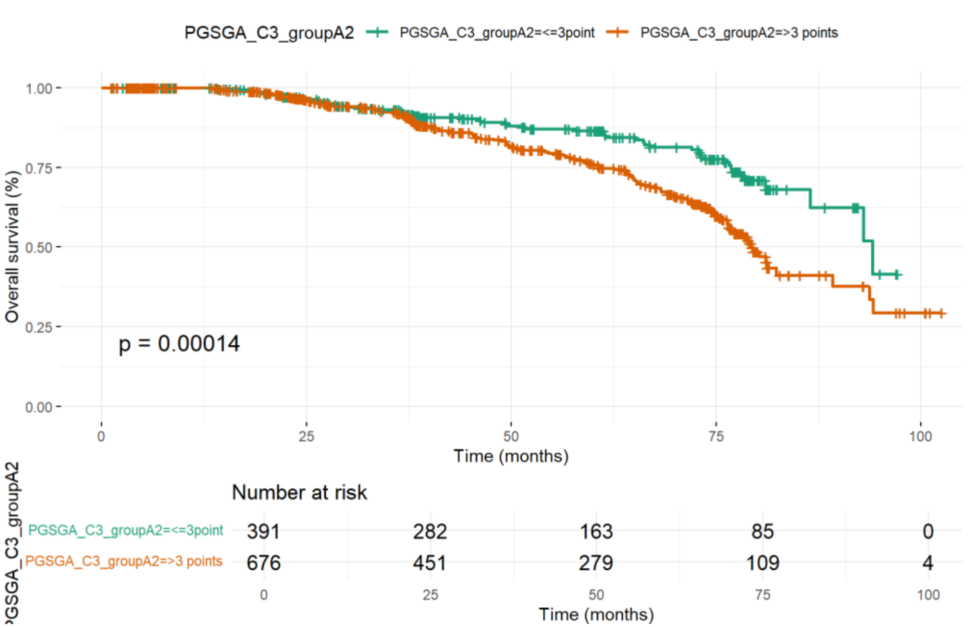

Figure S27 PGSGA_C3

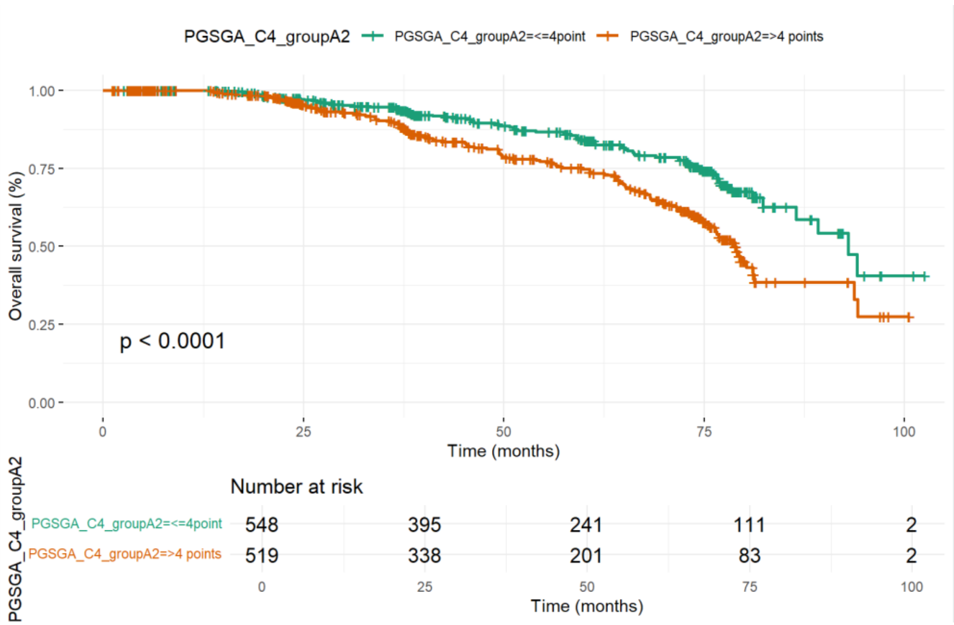

Figure S28 PGSGA_C4

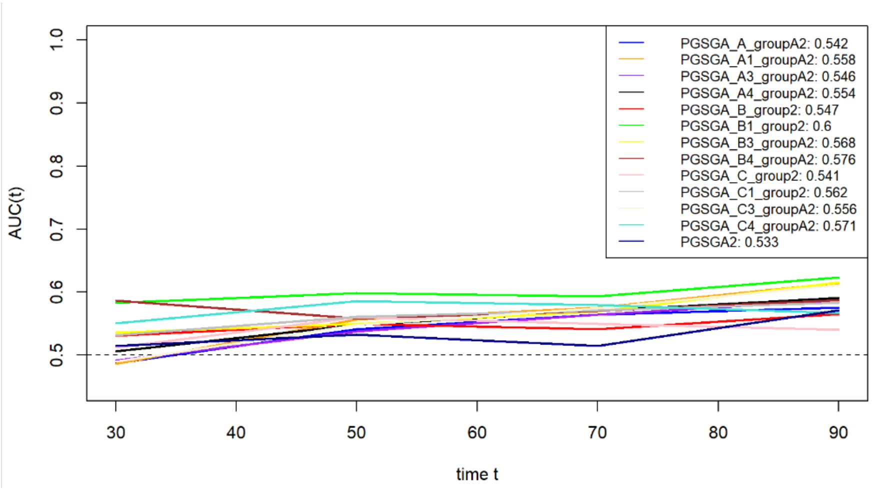

Figure S29 Binary Classification AUC

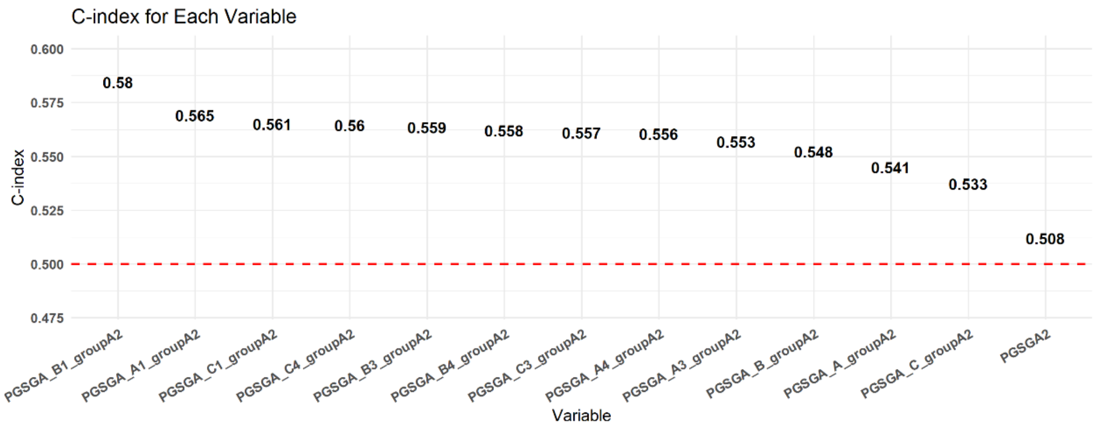

Figure S30 Binary Classification C-Index

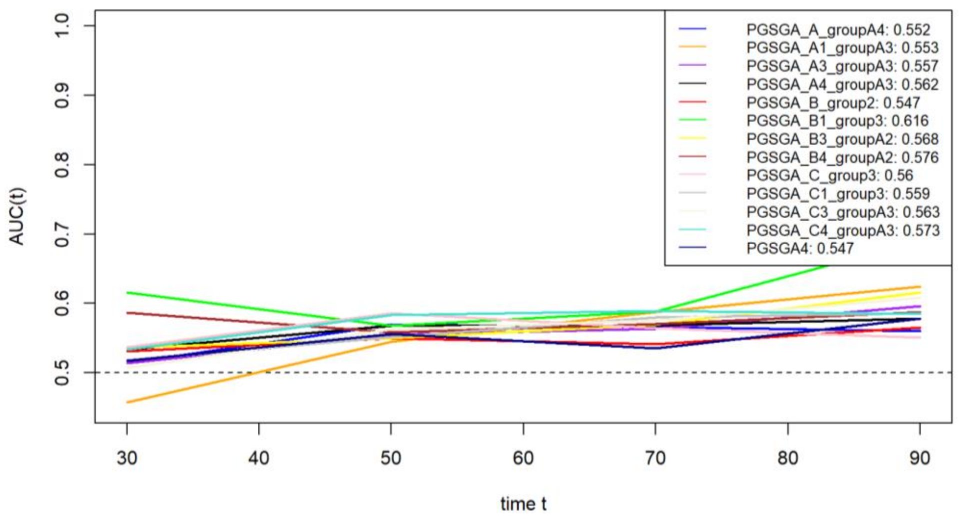

Figure S31 Four-Class Classification AUC

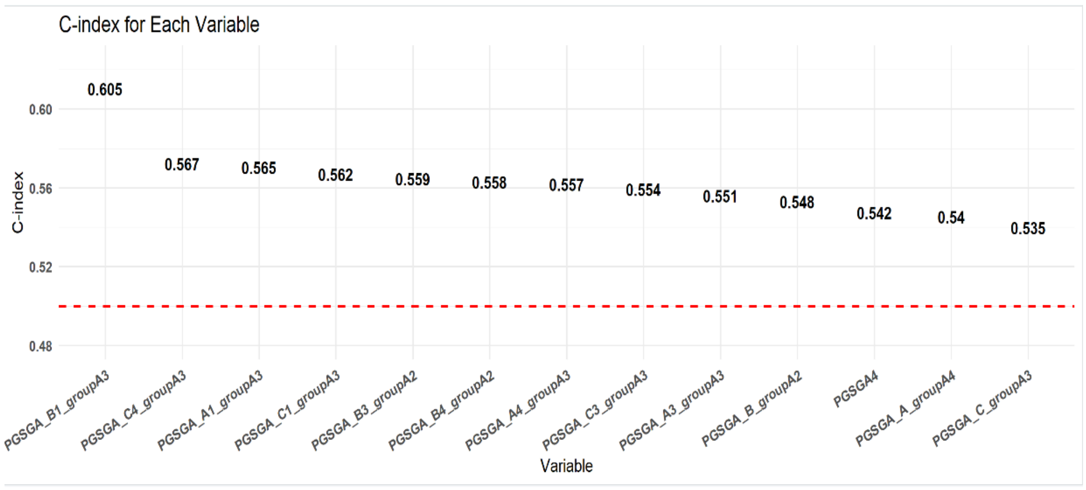

Figure S32 Four-Class Classification C-Index

Nutritional tools	Subgroup	β (95% CI)	P value
abPG-SGA	≥6 VS <6	4.95 (3.28–6.63)	<0.001
mPG-SGA	3-6 VS ≤ 2	1.44 (-0.67–3.55)	0.222
	≥7 VS ≤2	4.37 (2.37–6.38)	<0.001
PG-SGASF	4-8 VS ≤3	2.15 (0.24–4.06)	0.027
	≥9 VS ≤3	5.53 (3.52–7.53)	<0.001
PGSGA4	2-3 VS ≤1	-2.10 (-4.70–0.44)	0.102
	4-8 VS ≤1	0.79 (-1.71–3.07)	0.627
	≥9 VS ≤1	4.52 (1.83–6.56)	0.001
PGSGA3	4-8 VS ≤3	2.15 (0.32–3.97)	0.028
	≥9 VS ≤3	5.87 (3.80–7.94)	<0.001
PGSGA2	>4 VS ≤4	3.46 (1.84–5.08)	<0.001
PGSGA1	>1 VS ≤1	0.92 (-1.42–3.25)	0.441
GLIM	2 VS 1	4.15 (2.40–5.90)	<0.001
	3 VS 1	2.03 (-0.50–4.57)	0.121
scored-GLIM	2 VS 1	2.08 (-0.29–4.45)	0.078
	3 VS 1	4.25 (2.49–6.02)	<0.001
NRS-2002	2 VS 1	3.82 (2.21–5.43)	<0.001

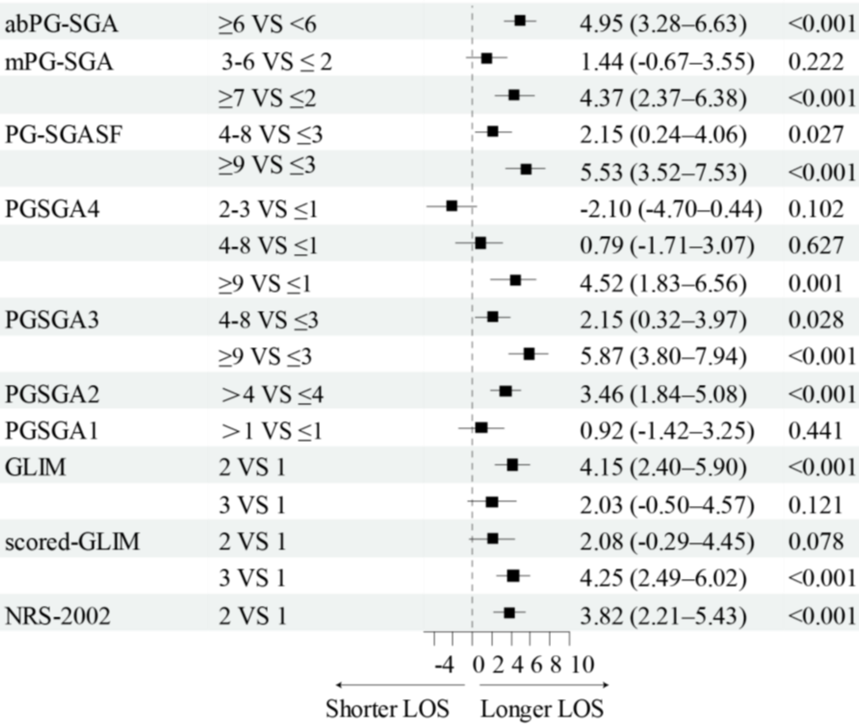

Figure S33 β（95%CI）of LOS and nutritional tools

Nutritional tools	Subgroup	β (95% CI)	P value
abPG-SGA	≥6 VS <6	8.75 (7.71 to 9.80)	<0.001
mPG-SGA	3–6 VS ≤2	1.46 (0.10 to 2.81)	0.035
	≥7 VS ≤2	7.87 (6.58 to 9.15)	<0.001
PG-SGA SF	4-8 VS ≤3	2.25 (1.07 to 3.44)	<0.001
	≥9 VS ≤3	10.22 (8.98 to 11.46)	<0.001
PGSGA4	2–3 VS ≤1	1.13 (-0.52 to 2.78)	0.178
	4–8 VS ≤1	3.89 (2.34 to 5.44)	<0.001
	≥9 VS ≤1	11.65 (9.98 to 13.31)	<0.001
PGSGA3	4–8 VS ≤3	3.16 (2.03 to 4.30)	<0.001
	≥9 VS ≤3	10.92 (9.63 to 12.20)	<0.001
PGSGA2	>4 VS ≤4	6.16 (5.09 to 7.22)	<0.001
PGSGA1	>1 VS ≤1	5.14 (3.56 to 6.72)	<0.001
GLIM	2 VS 1	3.42 (2.22 to 4.62)	<0.001
	3 VS 1	2.03 (0.29 to 3.77)	0.022
scored-GLIM	2 VS 1	1.47 (-0.15 to 3.10)	0.075
	3 VS 1	3.70 (2.49 to 4.91)	<0.001
NRS-2002	≥3 VS <3	3.40 (2.30 to 4.51)	<0.001

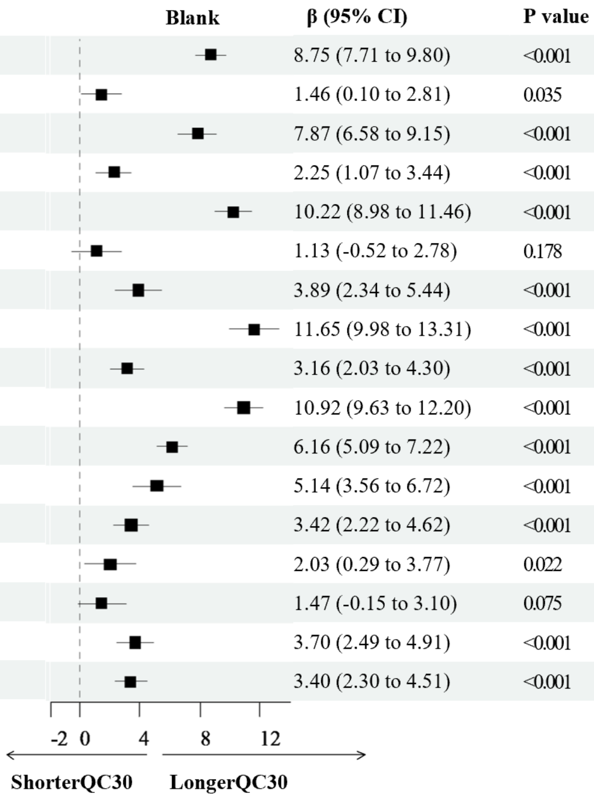

Figure S34 β（95%CI）of QC30 and nutritional tools

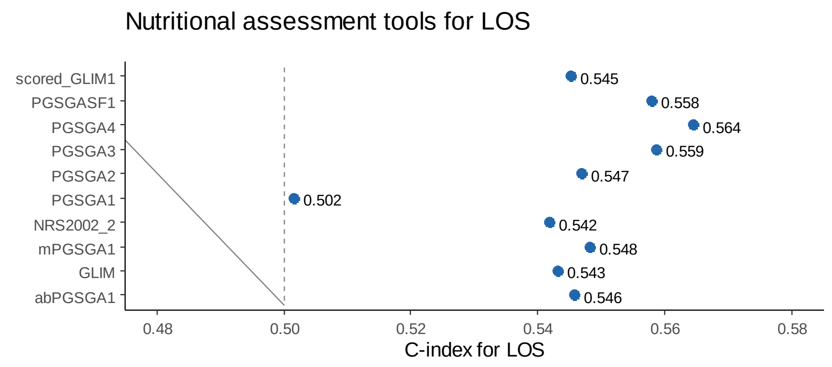

Figure S35 C-Index of LOS with nutritional tools

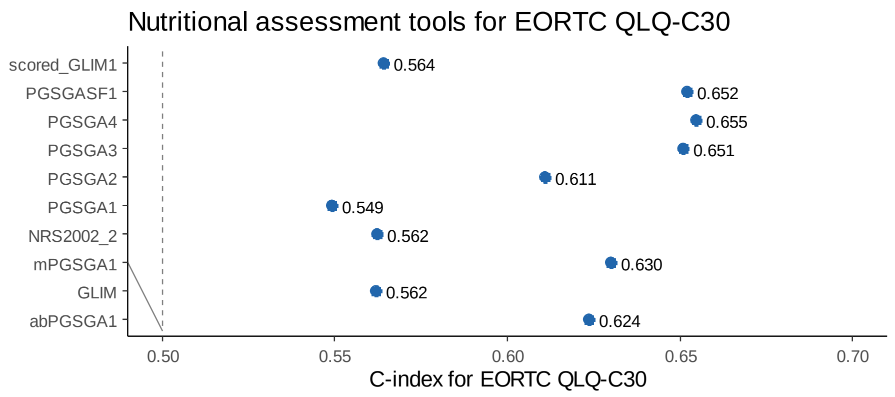

Figure S36 C-Index of QC30 with nutritional tools

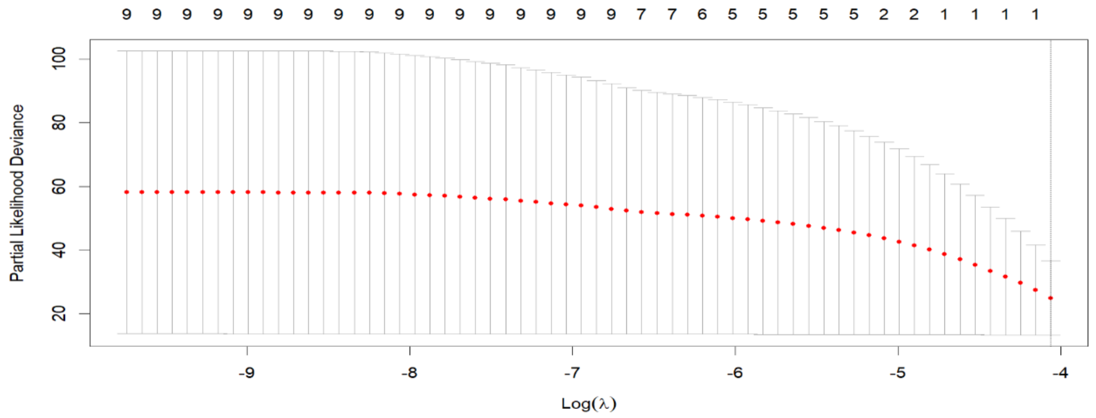

Figure S37 Lasso Regression: Hematological Parameters
